# Intracellular signaling pathway in dendritic cells and antigen transport pathway in vivo mediated by an OVA@DDAB/PLGA nano-vaccine

**DOI:** 10.1186/s12951-021-01116-8

**Published:** 2021-11-27

**Authors:** Shulan Han, Wenyan Ma, Dawei Jiang, Logan Sutherlin, Jing Zhang, Yu Lu, Nan Huo, Zhao Chen, Jonathan W. Engle, Yanping Wang, Xiaojie Xu, Lei Kang, Weibo Cai, Lianyan Wang

**Affiliations:** 1 Key Laboratory of Green Process and Engineering, State Key Laboratory of Biochemical Engineering, Institute of Process Engineering, Chinese Academy of Sciences, Beijing, 100190 People’s Republic of China; 2 University of Chinese Academy of Sciences, Beijing, 100049 People’s Republic of China; 3 School of Pharmaceutical Sciences, Jilin University, Changchun, 130021 People’s Republic of China; 4 Tianjin University of Science and Technology, Tianjin, 300222 People’s Republic of China; 5 Department of Nuclear Medicine, Union Hospital, Tongji Medical College, Huazhong University of Science and Technology, Wuhan, 430022 People’s Republic of China; 6 Departments of Radiology and Medical Physics, University of Wisconsin - Madison, Madison, WI 53705 USA; 7 Institute of Veterinary Immunology &Engineering, Jiangsu Academy of Agricultural Sciences, Nanjing, 210014 People’s Republic of China; 8 Department of Genetic Engineering Laboratory, Beijing Institute of Biotechnology, Beijing, 100850 People’s Republic of China; 9 Department of Nuclear Medicine, Peking University First Hospital, Beijing, 100034 People’s Republic of China

**Keywords:** DDAB/PLGA, Nano-vaccine, DCs activation, p38 signaling pathway, Antigen transport

## Abstract

**Background:**

Poly(D, L-lactic-co-glycolic acid) (PLGA) nanoparticles have potential applications as a vaccine adjuvant and delivery system due to its unique advantages as biodegradability and biocompatibility.

**Experimental:**

We fabricated cationic solid lipid nanoparticles using PLGA and dimethyl-dioctadecyl-ammonium bromide (DDAB), followed by loading of model antigen OVA (antigen ovalbumin, OVA_257-264_) to form an OVA@DDAB/PLGA nano-vaccine. And we investigated the intracellular signaling pathway in dendritic cells in vitro and antigen transport pathway and immune response in vivo mediated by an OVA@DDAB/PLGA nano-vaccine.

**Results:**

In vitro experiments revealed that the antigen uptake of BMDCs after nanovaccine incubation was two times higher than pure OVA or OVA@Al at 12 h. The BMDCs were well activated by p38 MAPK signaling pathway. Furthermore, the nano-vaccine induced antigen escape from lysosome into cytoplasm with 10 times increased cross-presentation activity than those of OVA or OVA@Al. Regarding the transport of antigen into draining lymph nodes (LNs), the nano-vaccine could rapidly transfer antigen to LNs by passive lymphatic drainage and active DC transport. The antigen^+^ cells in inguinal/popliteal LNs for the nano-vaccine were increased over two folds comparing to OVA@Al and OVA at 12 h. Moreover, the antigen of nano-vaccine stayed in LNs for over 7 days, germinal center formation over two folds higher than those of OVA@Al and OVA. After immunization, the nano-vaccine induced a much higher ratio of IgG2c/IgG1 than OVA@Al. It also effectively activated CD4^+^ T, CD8^+^ T and B cells for immune memory with a strong cellular response.

**Conclusion:**

These results indicated that DDAB/PLGA NP was a potent platform to improve vaccine immunogenicity by p38 signaling pathway in BMDCs, enhancing transport of antigens to LNs, and higher immunity response.

**Graphical Abstract:**

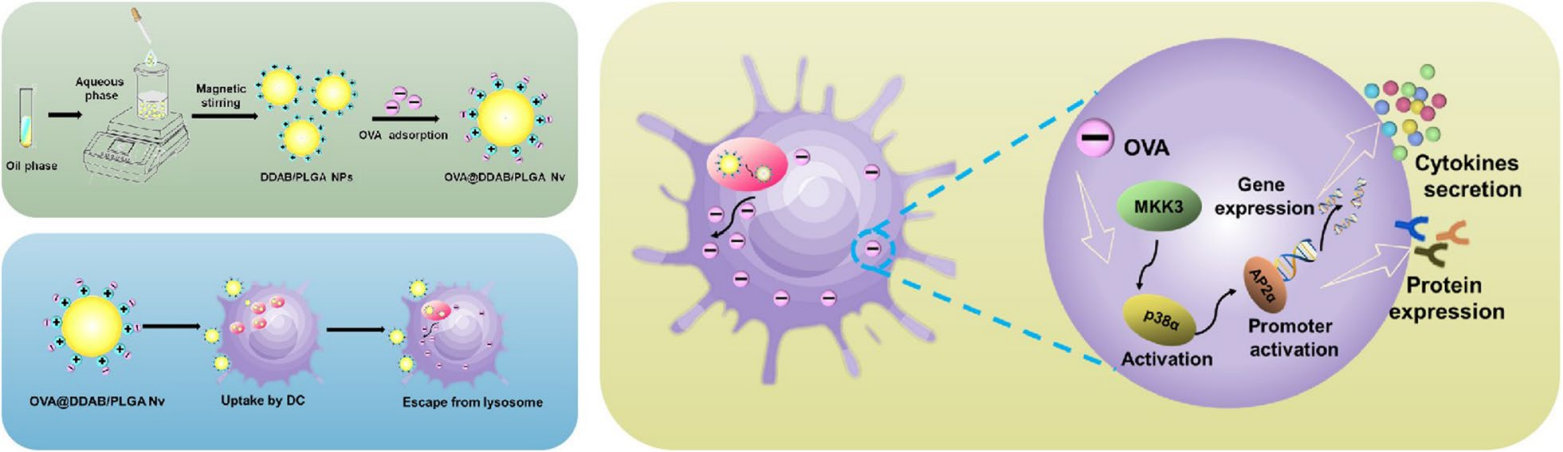

**Supplementary Information:**

The online version contains supplementary material available at 10.1186/s12951-021-01116-8.

## Introduction

Vaccination plays a crucial role in controlling the dissemination of virus and reducing morbidity and mortality [1]. Subunit vaccines of recombinant proteins and epitope peptides are emerging in interest as a safer alternative to traditional vaccines [2–4]. However, the ability of these vaccines to elicit a long-lasting and potent immune response is limited because their drawbacks as comparatively lower molecular weights and weaker immunogenicity [5]. The need for adjuvants is urgent and increasing. Al, the most common adjuvant licensed for human use, has been widely employed for about 90 years [6, 7]. Its application is limited by disadvantages such as side effects and anergy to cellular immunity, which drives the development of new delivery system and adjuvants for these subunit vaccines [8, 9].

Biodegradable nanoparticles are being investigated as a delivery vector for vaccines and adjuvant systems in recent years that can boost weak antigen effectiveness by enhancing antigen processing and/or as immune-potentiating adjuvants to induce or potentiate immune responses. These nanoparticles are made of biodegradable materials such as polysaccharides, proteins, fatty acids lipids and polymers [10–13]. A notable material is poly(D,L-lactic-co-glycolic acid) (PLGA) which has been approved to be used as pharmaceutical excipient by Food and Drug Administration (FDA). Studies have reported dual functionality of PLGA nanoparticles as both antigen delivery and adjuvant properties for enhancing immunity by promoting antigen engulfing by dendritic cells (DCs), activating and maturating DCs, and inducing effective immune response [14]. Compared to negative surface-charged nanoparticles, cationic ones can facilitate antigen adsorption and uptake by antigen-presenting cells (APCs) as DCs [15]. While cationic polymers such as chitosan, polylysine and polyethyleneimine have been employed to coat nanoparticles to enhance immune responses, concerns for cytotoxicity caused by plasma membrane instability and generation of reactive oxygen species (ROS) persist [16, 17].

Herein a relatively safe cationic surfactant dimethyl-dioctadecyl-ammonium bromide (DDAB) is chosen to fabricate positive surface-charged nanoparticles as DDAB/PLGA NPs. DDAB is commonly used to prepare cationic liposomes for regulating immunity and has been proven effective and safe by phase I clinical trials [18]. Then the model antigen ovalbumin (OVA_257-264_) is loaded onto the DDAB/PLGA NP by surface-adsorption to form an OVA@DDAB/PLGA nano-vaccine. While a number of nanoparticle-based vaccines showed improvement of antigen stability and immunogenicity, challenges still remain due to a lack of fundamental understanding regarding intracellular signaling pathway in DCs and in vivo behavior of nano-vaccines.

Nanoparticle-based vaccines has attracted extensive interest with a focus on how to deliver antigens more efficiently to APCs as DCs in order to induce their maturation and to enhance antigen presentation for activating immune response [19]. Further understanding of intracellular signaling pathway in DCs is crucial to develop efficacious nano-vaccines. Designing a safe and effective nano-vaccines also requires a strong understanding of their interaction with biological systems and their fate in vivo [1, 20]. A notable pathway is the nano-vaccines’ transport into the lymph nodes (LNs), where it further induces activation of B and T cells. Few studies about the antigen transport from injection site to LNs are reported due to the high resolution required to monitor the dynamic pathway of the small amount of antigen administered.

For this study the intracellular signaling pathway in BMDCs, the antigen transport to draining lymph nodes (LN) in vivo and subsequent immune response of the OVA@DDAB/PLGA nano-vaccine is systematically investigated. Understanding the interaction of nano-vaccine with the immune system is crucial for rational design and effective application of nano-vaccines.

## Materials and methods

### Materials and reagents

Poly (D, L-lactic-co-glycolic acid) (75:25 PLGA, Resomer® RG 752H, M_W_ 4A) was purchased from Lakeshore Biomaterials (Birmingham, AL, USA). Ovalbumin (OVA_257-264_) and dimethyl-dioctadecyl-ammonium bromide (DDAB) were obtained from Sigma (St, Louis, MO, USA). Methylene chloride (AR grade) and absolute ethyl alcohol (AR grade) were purchased from Beijing Chemical Reagent Company (Beijing, China). Concanavalin A, Roswell Park Memorial Institute (RPMI) 1640 medium, Dulbecco's modified Eagle medium (DMEM) medium, and fetal bovine serum (FBS) were supplied by Gibco (Grand Island, NY, USA). Alexa 635-phalloidin and Lysol-Tracker probes were purchased from Invitrogen (Grand Island, NY, USA). FITC was obtained from Sigma-Aldrich. Fluorochrome-labeled α-MHCI, α-MHC II, α-CD86, α-CD80, α-CD11c, α-CD69, α-CD44, α-CD62L, α-CD4, α-CD8 and α-CD19 antibodies were supplied by eBioscience (San Diego, CA, USA). Recombinant mouse GM-CSF and IL-4 were obtained from Peprotech (Rocky Hill, NJ, USA). Mouse cytokine ELISA kits were purchased from eBioscience. OVA derived (H-2 Kb, SIINFEKL) specific MHC I pentamers was purchased from ProImmune (Oxford, UK). p38 MAPK inhibitor SB203580 was purchased from Cell Signaling Technology (Danvers, MA, USA).

### Preparation of OVA@DDAB/PLGA nano-vaccine

**Fig. 1:**
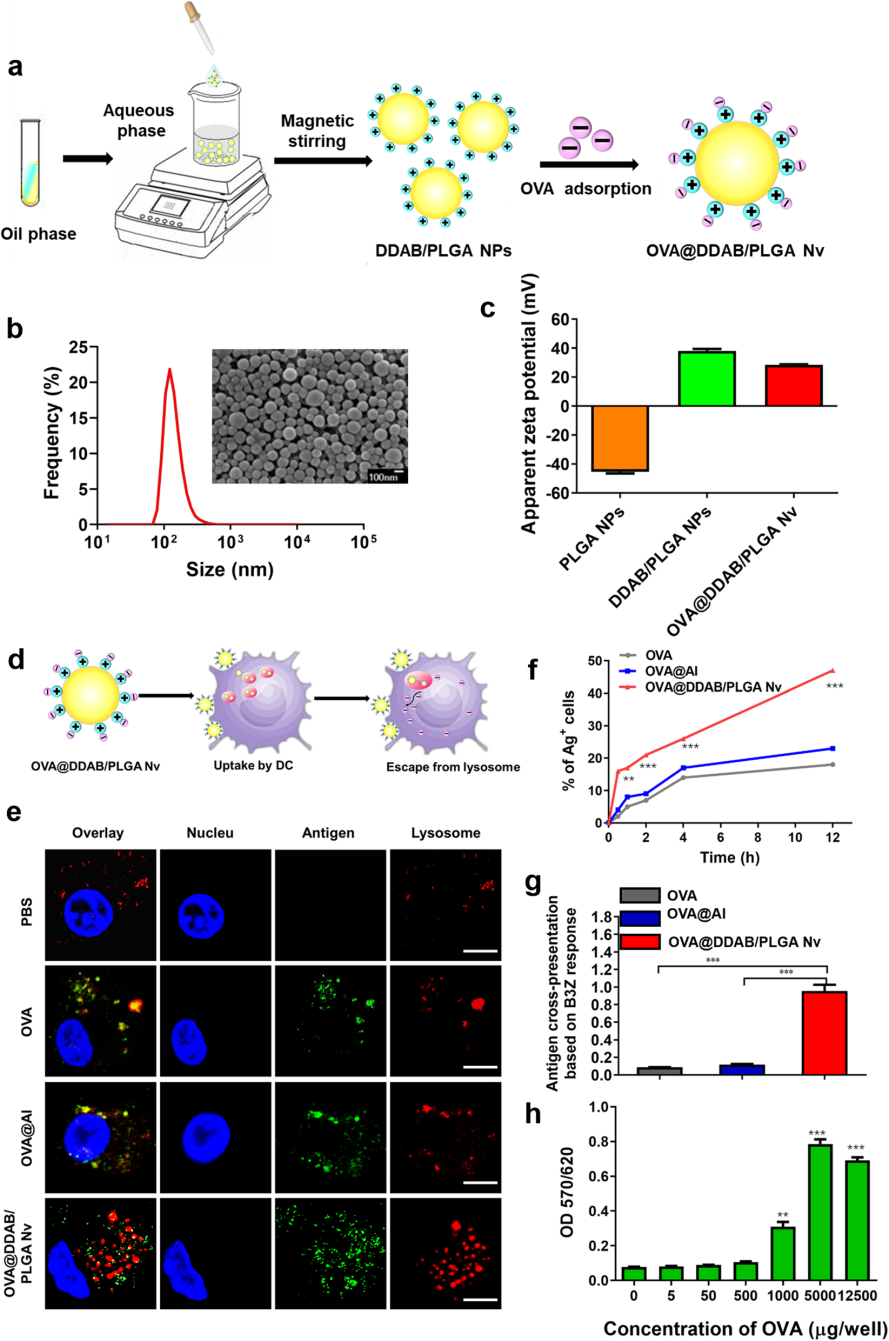
Preparation and characterization of the DDAB/PLGA Nano-vaccine (OVA@DDAB/PGA Nv). **a** Schematic illustration of the OVA@DDAB/PLGA Nv preparation. **b** SEM images and size distributions of OVA@DDAB/PLGA Nv, bar = 100 nm. **c** Zeta potential of PLGA NPs, DDAB/PLGA NPs and OVA@DDAB/PGA Nv in pH 7.4 PBS. **d** Illustration of OVA@DDAB/PLGA Nv uptake by DCs and escape from lysosome. **e** Antigen intracellular localization from pure antigen (OVA), antigen-loaded Al (OVA@Al), OVA@DDAB/PLGA Nv in BMDCs was observed by CLSM. Antigens were labeled by FITC (green points); lysosomes were labeled by Lyso-Tracker Red (red points); cell nucleus was labeled by DAPI (blue); bar = 20 μm. **f** Antigen uptake by BMDCs was measured by flow cytometry. **g** Different groups of vaccine adjuvant-based OVA (5 μg) were cross-presented by BMDCs and B3Z in vitro*.* **h** Different concentrations of free OVA (5, 50, 100, 1000, 5000, 12,500 μg) were cross-presented by BMDCs and B3Z in vitro. Cross-presentation of B3Z cells was determined by β-galactosidase activity using a colorimetric LacZ assay (absorbance 570/620 nm). Graphs shown represent results of three independent coculture experiments. Data are expressed as Mean ± STD (n = 3) (*p < 0.05; **p < 0.01; ***p < 0.001)

DDAB/PLGA nanoparticles were prepared using nanoprecipitation method previously developed in our laboratory (Fig. 1A). The 100 mg PLGA polymer and 30 mg DDAB were dissolved in 12 mL mixture solvent with acetone and anhydrous alcohol with volume ratio of 5:1. The solution was then mixed into 90 mL deionized water under magnetic bar stirring. Next, the suspension was continually stirred at rate of 500 rpm overnight to evaporate organic solvent in the solution. DDAB/PLGA nanoparticles were then collected by centrifugation at 20,000 × g for 15 min and washed three times in ultrapure water. The nanoparticles were then resuspended in an OVA antigen solution (250 μg/mL in PBS) and incubated for 3 h at room temperature while rotating to adsorb the OVA antigen, after which the product was lyophilized to obtain the OVA@DDAB/PLGA Nano-vaccine (OVA@DDAB/PLGA Nv) product.

### Characterization of OVA@DDAB/PLGA Nv

The morphology of the DDAB/PLGA nanoparticle was characterized with JSM-6700F scanning electron microscopy (SEM). Size distributions and zeta potentials were measured with Nano-ZS Zeta Sizer (Malvern Instruments Ltd., Malvern, UK). Concentrations of the OVA antigen were determined using Micro-BCA protein assay kit (Thermo Fisher, USA). The amounts of OVA antigen adsorbed to the DDAB/PLGA nanoparticles were calculated by subtracting the amount of OVA antigen remaining in the supernatant after absorption from the amount added into the system.

#### Cells viability assays

Cell viability was assessed using a colorimetric cell counting kit-8 (CCK-8) from Dojindo Laboratories (Kyoto, Japan). Cells were plated in 96-well plates at a density of 1 × 10^5^ cells/mL in 150 μL of growth medium per well. The wells were then treated with 50 μL of vaccine formulations with different antigen concentrations prepared by diluting a stock solution in growth medium. After 24 h, 10 μL CCK-8 reagent was added to each well and incubated at 37 °C for 4 h. Absorbance at 450 nm was measured, and the results were calculated from the ratio of the average OD450 values of wells containing NPs-stimulated cells to those containing only cells with medium.

#### Blood biomarker assay

Mice (BALB/c, female, 4–6 weeks) were intramuscularly injected with 100 μL (50 μL per hind leg) of PBS, OVA (Ag), OVA@Al, OVA@DDAB/PLGA-Nv (50 μg antigen per mouse per injection) on day 0, 14, and 28, then the sera were collected on day 35. Physiological conditions were evaluated by measuring the levels of aspartate aminotransferase (AST) for cardiac damage, alanine aminotransferase (ALT) for liver function, blood urea nitrogen (BUN) for nephrotoxicity, lactate dehydrogenase (LDH) for main organ injury, and alkaline phosphatase (ALP) for hepatobiliary damage with a Biochemical Autoanalyzer (TBA-40, Toshiba, Japan)[21].

### Antigen uptake and intracellular localization by bone marrow-derived dendritic cells (BMDCs) in vitro

BMDCs were differentiated from bone marrow cells using an established protocol [17]. In brief, bone marrow cells were isolated from C57BL/6 mouse (4–6 weeks) femurs and tibias that were cultured in RPMI medium 1640 supplemented with GM-CSF and IL-4 (10 and 50 ng/mL respectively) at 37 °C for 6 days after which the immature BMDCs were harvested for the follow-up experiments. For antigen uptake immature BMDCs were co-cultured with FITC-OVA or FITC-OVA@NP at 37 °C for 24 h. Cells were then washed twice with PBS (10 mM, pH 7.4) and stained with anti-CD11c antibody diluted with flow cytometry staining buffer (eBioscience) for BMDCs identification. The percentage of CD11c^+^ FITC-OVA^+^ cells was measured using flow cytometry (Beckman Coulter CyAnTM ADP) and analyzed using Summit software (version 4.3).

To evaluate antigen intracellular localization in BMDCs, immature BMDCs were plated onto a poly-D-lysine-coated petri dish for 4 h and then washed with PBS to remove non-adherent BMDCs. FITC-OVA (Ag), FITC-OVA@Al (Al), and FITC-OVA@NPs (OVA@DDAB/PLGA Nv) were added into the Petri dish. After incubation for 24 h, the cells were washed three times by PBS (10 mM, pH 7.4). The lysosomes were labelled with 75 nM LysoTracker®Red DND-99 (Molecular Probes-Invitrogen, CA, USA). BMDCs were further fixed in 100 μL 4% paraformaldehyde for 15 min. After that, the cell nuclei were labeled with DAPI for 20 min and the fluorescent images were obtained using confocal laser scanning microscopy (CLSM) (Leica, Germany).

### Detection of antigen presentation with a LacZ T cell hybridoma assay

Antigen cross-presentation was detected using a CD8^+^ T cell hybridoma B3Z cells line with β-galactosidase expression promoted by IL-2. B3Z cells (1 × 10^5^/well) were incubated with BMDCs (2 × 10^5^/well) in a 96-well plate. Cells were co-cultured with antigen of OVA or NP-adsorbed antigens and incubated at 37 °C and 5% CO_2_ for 18 h. Plates were then washed with PBS, and 100 μL of LacZ buffer (0.13% Nonidet P-40, 9 mM MgCl_2_, 0.15 mM chlorophenol red-β-D-galactopyranoside (Roche) in PBS) was added and incubated at 37 °C for 4 h. Absorbance was measured at 570/620 nm using a microplate reader (Tecan, Germany).

### Activation and maturation of BMDCs by OVA@DDAB/PLGA Nv in vitro

Immature BMDCs were stimulated with OVA (Ag), OVA-adsorbed Al (OVA@Al) or OVA-adsorbed DDAB/PLGA NPs (OVA@DDAB/PLGA Nv) for 16 h. Cells were then washed, collected, and labeled with fluorochrome-labeled antibodies against CD40, CD86 and MHC II diluted with flow cytometry staining buffer. The expression of CD40, CD86 and MHC II on BMDCs was measured by flow cytometry. Cytokines of IFN-γ, IL-1β, IL-22, TNF-α, and IL-6 in culture supernatants were also quantified using a Luminex multifactor detection kit (Merck-Millipore) according to the manufacturer's protocol and calculated using a five-parameter curve obtained from the absorbance values of standards provided by the manufacturer.

### Real-time quantitative PCR analysis

BMDCs were treated with OVA (Ag), OVA-adsorbed Al (OVA@Al), and OVA-adsorbed DDAB/PLGA NPs (OVA@DDAB/PLGA Nv), respectively, with a dosage of OVA 2.5 μg per well. Cells of each group were harvested after culturing for 24 h. RNA was extracted with TRIzol reagent (Invitrogen, USA). A reverse transcription reaction was performed using cDNA synthesis kit (TAKARA, Japan). mRNA expression of IL-1β, TNF-α, and IFN-γ was detected by ABI 7700 Sequence Detection System (PE Applied-Biosystems, Foster City, CA) using specific primers: (IL-1β, 5’-CCACAGGCTCACCCATACTTC-3’ (forward) and 5’-GGGATGTCCTAGGTGGTGACA-3’ (reverse); IFN-γ, AGAGCATGGAAACCCCGAAAC (forward) and TCAGATATTTCCAGTGGCCTG (reverse); TNF-α, CTGGAGCCATGGGTATGCA (forward) and AAGCACAAGCCGAGACTGCT (reverse)). Threshold cycle (Ct) was used to calculate the relative template quantity using β-actin as an internal control. The relative mRNA release was the fold changes was calculated by the Ct method and were relative to the basic gene expression.

### Western blot analysis

Immature BMDCs (2 × 10^6^) were exposed to OVA (Ag), OVA-adsorbed Al (Al) or OVA-adsorbed DDAB-PLGA NPs (DDAB-PLGA NVs) for different times and then exposed to DDAB-PLGA NVs with various nanoparticles concentrations for 12 h. Cells were washed by cold PBS and lysed in 50 μL RIPA buffer (20 mM Tris pH 7.4, 137 mM NaCl, 2 mM EDTA pH 7.4, 1% Triton, 25 mM β-glycerophosphate, 1 mM Na_3_VO_4_, 2 mM sodium pyrophosphate, 10% glycerol, 1 mM PMSF, 10 μg/mL aprotinin and 10 μg/mL leupeptin). Equal amounts of proteins were loaded onto 12.5% SDS-PAGE gel and transferred onto a NC membrane (Millipore). Membranes were then incubated with antibodies against the phospho-p38 MAPK (Thr180/Tyr182), phospho-AKT (Ser473) or phospho-ERK (Thr202/Tyr204), and total p38 MAPK, total AKT, or total ERK (all from Cell Signaling Technology, Danvers, MA, USA). Immunoreactive bands were detected by chemiluminescence (ECL solution, Amersham Biosciences).

### Co-immunoprecipitation

For detecting the interaction of endogenous p38α with MKK3, co-immunoprecipitation assays were conducted. Briefly, DCs cells treated with OVA@DDAB/PLGA Nv and blank group, lysed in 0.5 ml lysis buffer (50 mM Tris at pH 8.0, 150 mM NaCl, 0.25% NP-40, 1 mM DTT and protease inhibitor tablets from Roche), and immunoprecipitated with anti-p38α (Cell Signaling Technology) or control IgG (Millipore). After extensive washing with lysis buffer, the immuno-precipitates were resolved by SDS–PAGE and were analyzed with Western blot using the anti-MKK3 antibody (Cell Signaling Technology).

### Animals and immunization

All animal experiments were performed according to the Guide for the Care and Use of Laboratory Animals and approved by Experimental Animal Ethics Committee. Specific pathogen-free female BALB/c mice were purchased from Beijing Laboratory Animal Center and were kept in a pathogen-free facility.

### Positron emission tomography (PET) imaging and biodistribution of OVA@DDAB/PLGA Nv

OVA@DDAB/PLGA Nvs were radiolabeled with ^89^Zr for PET by the methods reported in literature [22]. The antigen OVA was modified with p-SCN-Bn-DFO (DFO) and radiolabeled with ^89^Zr. In brief, OVA was dissolved in 0.1 M sodium bicarbonate solution, and the pH value was adjusted to 7.0. DFO-DMSO solution at a concentration of 4 mg/mL was mixed into the OVA solution for 1 h under 37 ℃. Afterwards 0.1 M Na_2_CO_3_ solution was added to the ^89^Zr oxalic acid solution (< 5 mCi), the pH value was adjusted to 7.5–8.0 with 0.4 µM NaOH. Immediately, DFO-OVA was added to the ^89^Zr solution and the reaction was conducted at 37℃ for 60 min for labeling. The ^89^Zr-labeled products were further purified using a PD10 column (GE Healthcare). Radiochemical purity was determined using iTLC plates (Fisher Scientific), developed in 0.1 M citric acid (pH 5). For surface adsorption ^89^Zr-OVA was incubated with different concentration of DDAB-PLGA or Alumina hydroxide adjuvant(Al)for up to 3 h at room temperature and further used to PET imaging.

Female D57 mice (6–8 weeks) were used in PET imaging studies. Mice were anesthetized using isoflurane/O_2_ (2% v/v) before injection. Anesthetized mice were intramuscularly injected in the hind legs with ^89^Zr-OVA DDAB/PLGA Nv (4.44 − 5.55 MBq/120–150 μCi each mouse) in PBS (100 μL). After injection mice were scanned using an Inveon micro-PET/CT scanner (Siemens) at different time points (5 min, 2, 4, 6, 9 h, 1, 2, 4, 6, 8, 10, 12, and 14 d). For each mouse, twenty million coincidence events were collected and reconstructed using an Inveon Research 3D workstation (Siemens). Regions of interest (ROI) were drawn on lymph nodes (LNs) and spleen to calculate the biodistribution (%ID/g). Mice were euthanized by CO_2_ asphyxiation and organs and blood were harvested and wet weighed. The collected organs and blood were counted an auto gamma counter (PerkinElmer). The resulting counts of organs and blood were used to calculate the percentages of the injected dose per gram of tissue (%ID/g).

## The effect of OVA@DDAB/PLGA Nv for antigen uptake in LN-residing DCs

FITC-labeled OVA@DDAB/PLGA Nv or OVA@Al (OVA@DDAB/PLGA Nv, 100 μL/mice; OVA, 25 μg/mice) were intramuscularly injected in the hind legs of female BALB/c mice (6–8 weeks, n = 6). LNs were isolated 24 h after injection. LNs were mechanically isolated, pipetted, and filtered using 200 mesh cell strainers. Cells were blocked with anti-CD16/CD32 in FCS buffer for 10 min, followed by staining DCs (CD11c^+^) with anti-mouse antibodies against CD11c, then detected by flow cytometry to figure out the amount of OVA-positive DCs.

Immunohistochemistry and flow cytometry were employed to detect antigen content and germinal center (GC) in draining LNs. Three mice were sacrificed after injection at 14, 21, and 28 days. Lymph nodes were harvested and frozen in Tissue Tek-OTC® (Sakura, Torrance, CA, USA) compound and sectioned in 8 μm slices using a freezing microtome (Leica, Germany). They were then blocked, immunostained with anti-mouse GL-7 for identification of GC for 12 h. Finally, the slices were washed, incubated with secondary antibody, and fixed. After processing, all stained sections were measured by the automatic multispectral imaging system (PerkinElmer Vectra II). In addition, the lymph nodes from the other three mice were grounded into cell suspensions for further analysis by flow cytometry. The anti-mouse GL-7 and anti-mouse B220 were employed to identify the GC, and the anti-mouse CXCR5, anti-mouse PD-1 and anti-mouse CD4 were using for identified the T follicular helper cell (TFH).

### The maturation and activation of dendritic cells in secondary lymphoid organs

Different vaccines were intramuscularly injected in the hind legs of female BALB/c mice (6–8 weeks, n = 6). The popliteal LNs and inguinal LNs were isolated at 3, 6, 12, 24, 48, 72 h and 7 days after injection. LNs were mechanically isolated and processed into a single cell suspension. Cells were stained with fluorescence-labelled anti-mouse antibodies against CD11c, CD86, MHC I and MHC II and determined by flow cytometry.

### Serum IgG and subclasses as IgG1 and IgG2 were determined by ELISA

Mice (6–8 weeks of age, n = 6/group) were intramuscularly immunized with 100 μL suspensions of formulations (OVA, OVA@Al, OVA@DDAB/PLGA Nv) on days 0, 14 and 28. After the first immunization on day 14, 21, 28, and 35, sera were collected from the orbital venous plexus of mice for antibody analysis. On day 35, mice were sacrificed to collect spleens for immunological tests. Antigen-specific IgG and IgG subclasses were detected quantitatively with previously established protocols [21]. Briefly, ELISA plates were coated with 5 μg of OVA per well in carbonate buffer (0.05 M, pH 9.6) for 24 h at 4 °C. Then, plates were washed four times with 0.01 M PBS containing 0.05% (m/v) Tween 20 (PBST) and then blocked with 2% (m/v) BSA in PBST for 1 h at 37 °C. After washing another four times, appropriate sera dilutions (100 μL/well) were added with a twofold dilution and incubated for 40 min. Afterwards, plates were then washed with PBST and stained with 100 μL of HRP-conjugated anti-mouse antibodies against IgG, IgG1 and IgG2 (diluted 1:10,000) for 30 min. The ELISA plates were washed again, and 200 μL of TMB substrate was pipetted into all wells and incubated at room temperature for 20 min. The reaction was quenched by adding 50 μL of 2 M H_2_SO_4_ and OD_450_ was measured and antibody titers were described as the reciprocal sample dilution corresponding to twice higher OD value than that of the negative sera.

### Cytokine measurements in splenocytes with ELISA

Afterwards the harvested spleens were then grounded through a 200-mesh cell strainer. Erythrocytes were lysed by 0.9% ammonium chloride, and splenocytes were washed three times with RPMI 1640. Splenocytes (1 × 10^6^) were cultured with OVA (5 μg/well) or concanavalin A (1 mg/mL) as a positive control at 37 °C, 5% CO_2_, and 95% humidity. Supernatants were harvested at 72 h to measure cytokines of IL-4, IL-6, IFN-γ, TNF-α and Granzyme B using ELISA kits according to the manufacturer's protocol (eBioscience). Cytokines in supernatants were calculated using a five-parameter curve obtained from the absorbance values of standards provided by the manufacturer.

### Evaluation of lymphocyte activation and T cell response by flow cytometry

The effect of OVA@DDAB/PLGA Nv on lymphocyte activation, memory T cell response, and antigen-specific CD8^+^ T cell response were evaluated by flow cytometry. Mice (n = 6/group) were intramuscularly vaccinated three times over a 2-week period. On day 35 after the first immunization, mice were euthanized and splenocytes were harvested and stimulated with OVA (OVA, 5 μg/well) in vitro. Splenocytes were then cultured (1 × 10^6^ cells/well) for 72 h in a 37 °C humidified incubator with 5% CO_2_. After washing with PBS, cells were stained with fluorescent-labeled anti-mouse antibodies against CD4, CD8, CD19, CD69, CD44, and CD62L (eBioscience). Flow cytometry was used to measure activated lymphocytes (CD69^+^), effector memory T cells (CD44^hi^ CD62L^low^), central memory T cells (CD44^hi^ CD62L^hi^), and antigen-specific CD8^+^ T cells (CD8^+^ CD69^+^). Data analysis was performed using Summit software.

### Activation of cytotoxic lymphocyte (CTL)

Splenocytes were seeded on 24-well plates with 1 × 10^6^ cells/well. The splenocytes were stimulated with OVA (5 μg/mL) and cultured at 37 ℃ with 5% CO_2_ for 72 h in vitro. Cells were collected and washed twice with PBS, then labeled with fluorescent-labeled antibodies (anti-FITC-CD8a, anti-APC-FasL, anti-PE-CD107a, anti-PE-cy7-perforin), and analyzed by flow cytometry. Summit software was used for data analysis.

### Statistical analysis

All results were expressed as the means ± standard error of the mean (SEM). Statistical analysis was performed using GraphPad Prism 5.0 software (San Diego, CA, USA). Differences between two groups were tested using an unpaired, two-tailed Student’s t-test. Differences among multiple groups were tested with one-way ANOVA followed by Tukey’s multiple comparison. Significant differences between groups were expressed as follows: *p < 0.05, **p < 0.01, or ***p < 0.001, ****p < 0.0001.

## Results and discussion

### Characterization of OVA@DDAB/PLGA Nv

DDAB/PLGA nanoparticles were successfully fabricated by nanoprecipitation method DDAB, as a cationic lipid material, endowed the NPs with positive surface charge. The positively charged surface of the DDAB/PLGA NPs enabled the negatively charged antigen of OVA to easily adsorb to the surface via electrostatic interaction to obtain OVA@DDAB/PLGA Nv (Fig. 1a). The OVA antigen adsorption efficiency was determined using Micro-BCA protein assay kit. The adsorption efficiency of OVA was more than 90% (OVA 235 μg/mg). Moreover, the morphology of OVA@DDAB/PLGA Nv was characterized using a scanning electron microscope (SEM), which showed a spherical structure (Fig. 1b). The particle size of OVA@DDAB/PLGA Nv was 123.1 ± 5.7 nm with a PDI of 0.021 ± 0.006, which was only 0.3 ± 0.1 nm larger than DDAB/PLGA NPs (Additional file 1: Table S1). This suggests that the PLGA NPs demonstrated a negative surface charge of − 43.7 ± 0.4, while the DDAB/PLGA NPs showed a positive surface charge of + 39.5 ± 0.2 (Additional file 1: Table S1). The DDAB/PLGA NPs with OVA antigen adsorption still maintained a positive surface charge of 28.7 ± 0.7 mV (Fig. 1c, Additional file 1: Table S1), which is favorable for enhancing cell uptake.

### OVA@DDAB/PLGA Nv promoted antigen cross-presentation in vitro

Dendritic cells (DCs), the most potent APCs, are necessary for inducing protective immunity [23]. After being taken up by DCs, the intracellular fate of antigens (including intracellular localization and presentation) dramatically influenced the magnitude and quality of the immune response. The safety of OVA@DDAB/PLGA Nv for BMDCs in vitro was confirmed using a CCK-8 kit. The negligible cytotoxicity of OVA@DDAB/PLGA Nv towards BMDCs indicated biocompatibility of the as-prepared OVA@DDAB/PLGA Nv (Additional file 1: Fig. S1). Intracellular trafficking of internalized nano-vaccines was then investigated via localization of different antigen-loaded vaccines (labeled with FITC-OVA in green) in lysosomes, the key intracellular organelles during antigen processing and presentation (labeled with Lyso-Tracker in red) (OVA: Ag; FITC: fluorescein isothiocyanate). Activating the CD8^+^ T cells and eliciting a robust cytotoxic T lymphocyte (CTL) response requires efficient exogenous endosomal escape of the antigens for presentation (Fig. 1d) [24]. Compared to pure antigen (OVA) and Al (OVA@Al) groups, the DDAB-PLGA nanoparticle delivery system (OVA@DDAB/PLGA Nv) significantly promoted antigen uptake by BMDCs, and most antigens resided in the cytoplasm (Fig. 1e). The proportion of antigen uptake by BMDC cells increased over time (Fig. 1f). At 12 h, OVA@DDAB/PLGA Nv maintained the highest uptake rate of 48%, two times more than OVA and OVA@Al (Fig. 1f). This was likely due to the positive surface charge of OVA@DDAB/PLGA Nv, which facilitated interactions between the nano-vaccines and cell membrane of BMDCs. The smaller size of OVA@DDAB/PLGA Nv was easily recognized and ingested by BMDCs, further enhance antigen uptake by BMDCs. This result suggests that the OVA@DDAB/PLGA Nv could promote antigen escape from the lysosome and further induce a stronger CTL immune response.

After confirming that the OVA@DDAB/PLGA Nv could be efficiently captured by BMDCs and escaped from the lysosome into the cytoplasm, we further analyzed their capacity to cross-present antigens in a B3Z cell line. B3Z, a SIINFEKL-specific CD8^+^ T cell hybridoma line, could be activated by SIINFEKL, which was the final peptide of DDAB-PLGA-based antigens cross-presented by BMDCs [21]. Cross-presentation was evaluated by measuring the activation of B3Z with a LacZ method. After co-culturing BMDCs and B3Z with OVA@DDAB/PLGA Nv (5 μg OVA/well), OVA@DDAB/PLGA Nv enhanced cross-presentation of OVA. Compared to pure antigen (OVA) and Al-adjuvanted vaccine (OVA@Al), OVA@DDAB/PLGA Nv showed a tenfold improvement in OVA cross-presentation at the same antigen dose (p < 0.001) (Fig. 1g). To determine if the OVA@DDAB/PLGA Nv promoted antigen cross-presentation processing, we compared its cross-presentation to the cross-presentation of different concentration of soluble OVA. It was found that OVA solution required as high as 1000 times antigen dosage of 5000 μg/well to achieve similar cross-presentation level of OVA@DDAB/PLGA NV (Fig. 1h). These results confirmed that the antigen-loaded nano-vaccines played an essential role in the rapid uptake and delivery of antigens for cross-presentation.

### OVA@DDAB/PLGA Nv promoted BMDCs activation and maturation in vitro

**Fig. 2:**
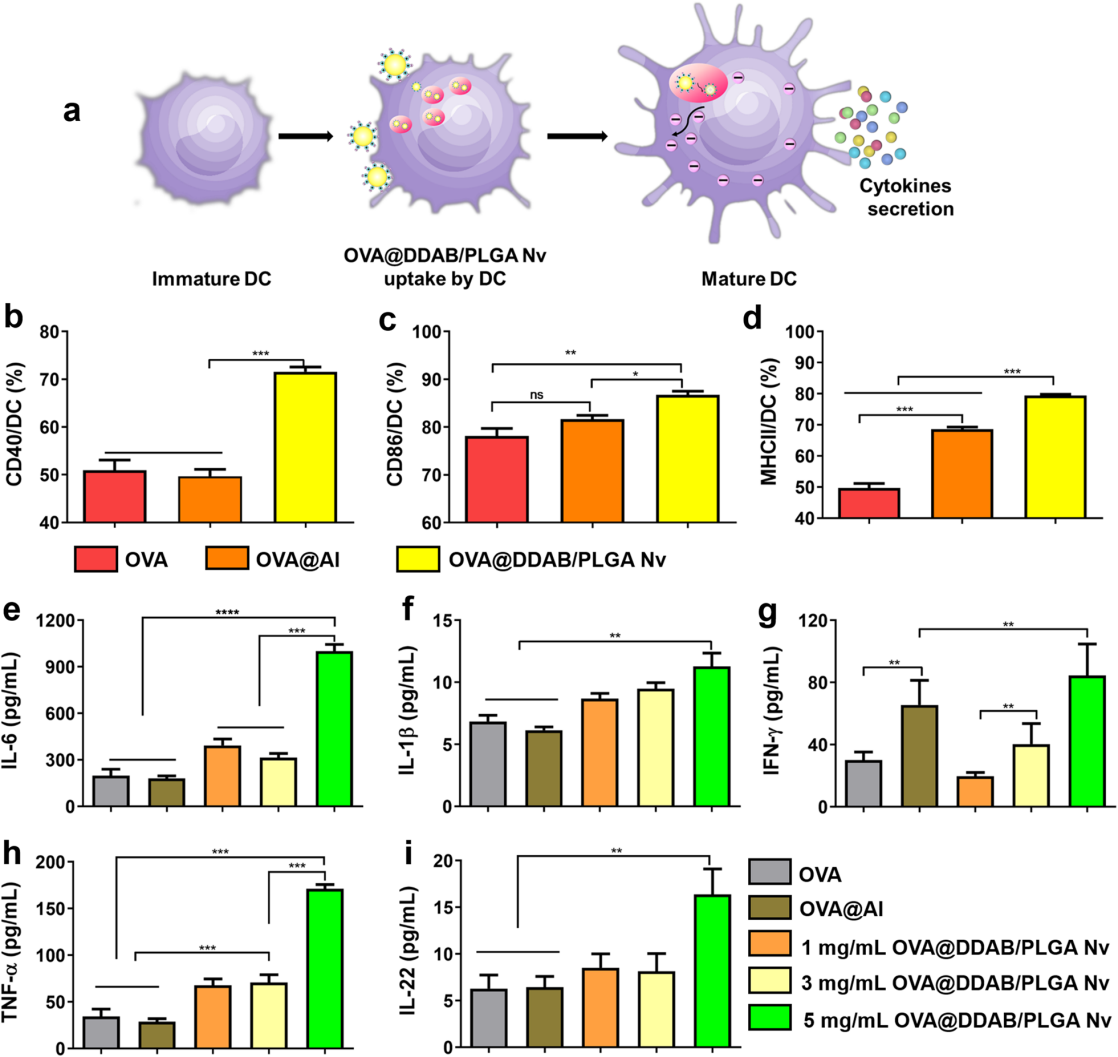
Expression of the co-stimulatory molecules and cytokine from DCs stimulated with different formulations. **a** Illustration of OVA@DDAB/PLGA Nv uptake by DCs and promoted DCs activation and maturation. **b**, **c**, **d** Percentages of CD40^+^ CD11c^+^ (**b**), CD86^+^ CD11c^+^ (**c**) and MHC II^+^ CD11c^+^ (**d**) cells were analyzed by flow cytometry. **e**, **f**, **g**, **h**, **i** Cytokine (IL-6, IL-1β, IFN-γ, TNF-α, and IL-22) secretion of DCs stimulated with different formulations for 24 h were measured by Luminex kit (Merck-Millipore). Graphs shown represent results of three independent experiments. Data are expressed as Mean ± STD (n = 3) (*p < 0.05; **p < 0.01; ***p < 0.001; ****p < 0.0001)

After antigen processing and presentation, DCs expressed activation surface and co-stimulatory markers and secreted cytokine to prime T cells, thereby inducing antigen-specific immune responses [23] (Fig. 2a). The expression of co-stimulatory markers CD40, CD86 and surface activation marker MHC II was significantly up-regulated by OVA@DDAB/PLGA Nv, compared with OVA and OVA@Al (Fig. 2b–d and Additional file 1: Fig. S2b–d; for CD40 and MHC II, *P* < 0.001; for CD86, compared to OVA, *P* < 0.01, compared to OVA@Al, *P* < 0.05). These results indicated that the OVA@DDAB/PLGA Nv promoted BMDCs activation compared to OVA and OVA@Al. Next, we tested whether OVA@DDAB/PLGA Nv also promoted cytokine secretion during DCs maturation. We detected and analyzed cytokines with stimulation of different formulations such as OVA, OVA@Al, and various concentrations of OVA@DDAB/PLGA Nv by Luminex kit (Merck-Millipore). OVA@DDAB/PLGA Nv induced greater production of proinflammatory cytokines IL-6 and IL-1β than OVA and OVA@Al groups (Fig. 2e, f). Secretion of Th1-polarizing cytokines IFN-γ and TNF-α followed the trends of OVA@DDAB/PLGA Nv which had an enhanced effect on maturation (Fig. 2g, h). Results indicate that 5 mg/mL of OVA@DDAB/PLGA Nv promoted cytokines secretion of IL-6, IL-1β, IFN-γ, TNF-α and IL-22, which was significantly higher than OVA and OVA@Al groups (Fig. 2e–i; for IL-6, *P* < 0.0001; for TNF-α, *P* < 0.001; for IL-1β, IFN-γ and IL-22, *P* < 0.01). Production cytokines IFN-γ, TNF-α and proinflammatory cytokines IL-1β, IL-6, and IL-22 were both found to be concentration-dependent (Fig. 2e–i). These results indicated that OVA@DDAB/PLGA Nv could induce DCs maturation and activation, which would product cytokines for further activating CD8^+^ T cells and CD4^+^ T cells.

### OVA@DDAB/PLGA Nv enhanced activation of BMDCs mainly by activating p38 MAPK signaling pathway

**Fig. 3:**
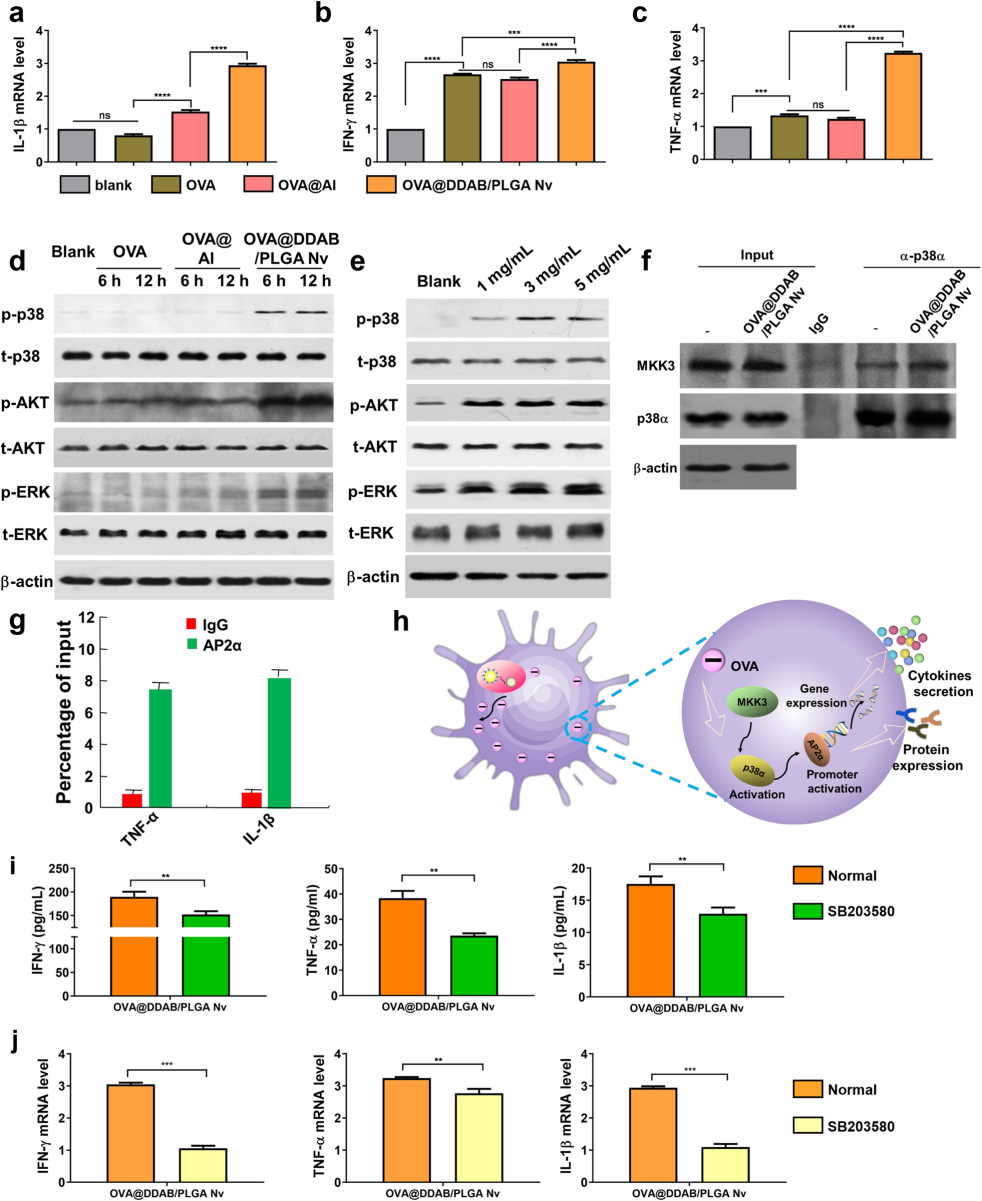
Cytokine mRNA expression and activation of relevant signal pathway profiles elicited by different vaccine adjuvants for BMDCs. **a**, **b**, **c** The cytokine (IL-1β, IFN-γ and TNF-α) mRNA expression from BMDCs was analyzed by RT-qPCR. **d** The changes of p38 MAPK, p-AKT and p-ERK phosphorylation after being stimulated with different formulation DCs for 6 h and 12 h. **e** Change of the p38 MAPK, p-AKT and p-ERK phosphorylation level by stimulated DCs with different concentration of DDAB-PLGA Nv. **f** DDAB-PLGA Nv increased the binding of MKK3 to its substrate p38α. **g** The ability of AP2α to recruit TNF-α and IL-1β promoters in the P38 pathway. **h** Illustration of how OVA@DDAB/PLGA Nv promoted DCs activation and maturation via the p38 MAPK signaling pathway. **i** After treatment with inhibitors of p38 (SB203580), the change of OVA@DDAB/PLGA Nv induced cytokine secretion from BMDCs. **j** After treatment with inhibitors of p38 (SB203580), the change of OVA@DDAB/PLGA Nv induced cytokine mRNA expression from BMDCs. Data are expressed as Mean ± STD (n = 6). (*p < 0.05; **p < 0.01; ***p < 0.001; ****p < 0.0001)

Then, we employed the ingenuity pathway analysis (IPA) system to explore the pathways that activate DCs by OVA@DDAB/PLGA Nv. First, the mRNA levels of cytokines (IL-1β, IFN-γ and TNF-α) were determined by RT-qPCR. OVA@DDAB/PLGA Nv significantly increased the mRNA levels of IL-1β, IFN-γ and TNF-α from DCs compared with those of OVA and OVA@Al groups (Fig. 3a–c; for IL-1β, *P* < 0.0001; for IFN-γ, *P* < 0.001; for TNF-α, *P* < 0.0001). Next, we focused on the signaling pathways related to the above three cytokines. MAPKs pathway including ERK1/2 and p38 MAPK and AKT pathways, which play an important role in the release of pro-inflammatory cytokines [25, 26]. Therefore, we tested the signaling pathway (MAPKs or AKT) of DCs activation induced by OVA@DDAB/PLGA Nv. The results indicated that the phosphorylation level of p38 significantly increased in the OVA@DDAB/PLGA Nv group while it had little effect in the OVA and OVA@Al groups (Fig. 3d and Additional file 1: Fig. S3a). The phosphorylation levels of AKT and ERK meanwhile also increased a little for the OVA@DDAB/PLGA Nv group as shown in the quantified data of Figure S3a. Furthermore, the ratio of p-p38 expression to t-p38 expression in OVA@DDAB/PLGA NV treatment was 10 folds more likely than the control group, while the ratio of p-ART and p-ERK to t-Akt and t-ERK was only as 1–2 folds as that of the control group. Therefore, we concluded that OVA@DDAB/PLGA NV mainly activated DC cells by activating the P38 signaling pathway, followed by p-AKT and p-ERK signaling pathways, and the latter was not the main activating pathway. Next, the activation of this signaling pathway in DCs treated with different concentrations of OVA@DDAB/PLGA Nv was detected. The p-p38 level increased markedly with increase of the dose of OVA@DDAB/PLGA Nv, indicating that the OVA@DDAB/PLGA Nv activated DCs by provoking the p38 MAPK signaling pathway (Fig. 3e and Fig.S3b). The mitogen-activated protein kinase 3 (MKK3) is the upstream and critical kinase of the p38 MAPK signaling pathway. To further investigate the mechanism of how the p38 MAPK signaling pathway was activated by the OVA@DDAB/PLGA Nv, we performed co-immunoprecipitation assays to evaluate the effect of OVA@DDAB/PLGA Nv on the interaction between kinase MKK3 and its substrate p38α. OVA@DDAB/PLGA Nv enhanced the interaction of MKK3 and p38α (Fig. 3f and Additional file 1: Fig. S3c) and increased the ability of AP2αto recruit the promoters of TNF-α and IL-1β in the p38 pathway (Fig. 3g). Taken together, activation of p38 signaling pathway plays a key role in inducing DCs maturation and cytokine secretion by OVA@DDAB/PLGA Nv (Fig. 3h).

### Inhibition of the p38 MAPK signaling pathway prevented the activation and maturation of BMDCs induced by OVA@DDAB/PLGA Nv

To further investigate the role of p38 MAPK signaling pathway in DCs maturation, we utilized the p38 MAPK-specific inhibitor, SB203580 [27] to determine whether inhibition of the p38 MAPK signaling pathway affected activation and maturation of BMDCs induced by OVA@DDAB/PLGA Nv. The results showed that SB203580 greatly down-regulated IFN-γ, TNF-α and IL-1β secretion induced by OVA@DDAB/PLGA Nv (Fig. 3i; *P* < 0.01). Moreover, the SB203580 reduced the mRNA expression levels of IFN-γ, TNF-α and IL-1β that induced by OVA@DDAB/PLGA Nv (Fig. 3j; for IFN-γ and IL-1β mRNA levels, *P* < 0.001; for TNF-α, *P* < 0.01). These results further demonstrated that OVA@DDAB/PLGA Nv activated BMDCs through the p38 signaling pathway.

### PET imaging and biodistribution study

**Fig. 4:**
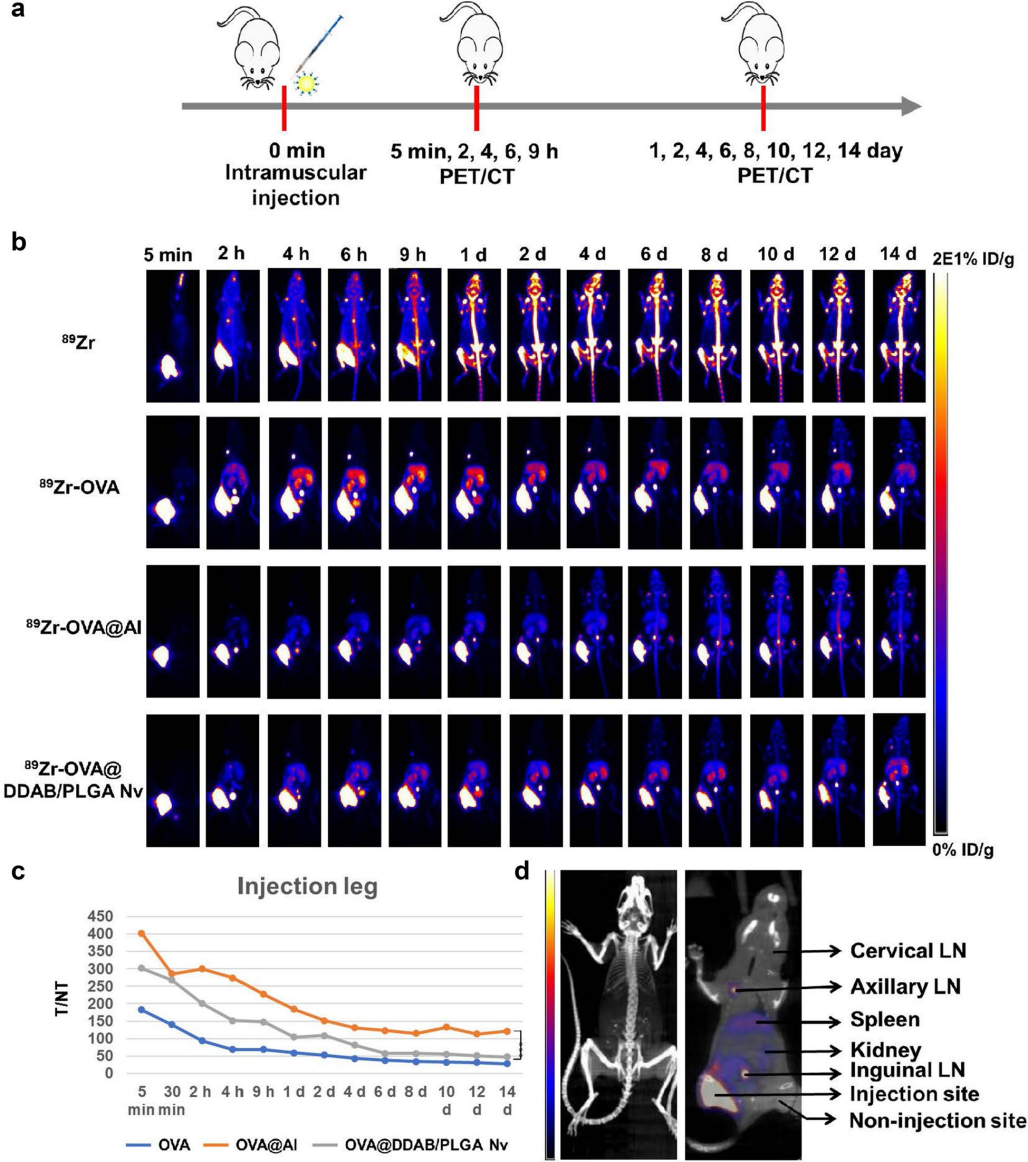
The mice PET/CT imaging showed the antigen retention at injection site and biodistribution in vivo. **a** Experimental design to evaluate the transport behavior of various vaccines. **b** The mice SPECT imaging of different ^89^Zr-OVA-loaded formulations. **c** Half quantitative T/N of antigen retaining at injection sites. T/NT: the ratio of the injection site (T) to the non-injection site (NT) of the radioactive semi-quantitation. **d** The diagram of internal organs in mice

Since the OVA@DDAB/PLGA Nv exhibit prominent adjuvant effect in promoting antigen presentation in vitro, we further explored the antigen transport and biodistribution in vivo. As a vaccine formulation, the antigen dose is usually low, which makes it difficult to quantify antigen transport by common techniques such as fluorescence labeling. We overcome this hurdle with PET imaging because it provides highly sensitive signal to trace and quantify radiolabeled probes [28]. Small animal PET imaging was used to track the transport behavior of OVA@DDAB/PLGA Nv after radiolabeling with ^89^Zr, allowing us to monitor the antigen retention at the injection site and the migration to lymph nodes. ^89^Zr-OVA, ^89^Zr-OVA@Al, and ^89^Zr-OVA@DDAB/PLGA Nv were prepared to detect their transport behavior. Biodistribution studies were then used to verify the imaging data (Fig. 4a).

The labeling process was completed within 50 min with an average yield of 62.88 ± 10.45% (n = 3). Instant thin-layer chromatography (ITLC) determined the radioactivity peak shift value of the product to be 0.242 and the ^89^Zr ion elution ratio shift value to be 0.325. The chemical purity of product was greater than 95%. The radiostability of ^89^Zr-DFO-OVA (^89^Zr-OVA) in room temperature, serum and saline were monitored over the course of two weeks. The radiostability of ^89^Zr-OVA at room temperature, saline (37 ℃) and serum (37 ℃) all remained over 75% during that time frame (Additional file 1: Fig. S4). Notably the radiostability of ^89^Zr-OVA in serum was higher than its stability in the room temperature and saline. These results indicated that ^89^Zr-OVA could be used for quantitative and qualitative research in subsequent dynamic antigen transporting processes in vivo.

The dynamic changes of radioactive distribution at the injection site and different organ were imaged by PET/CT imaging and region of interest (ROI) analysis of the collected images (Fig. 4a). As shown in PET images, the Al-adjuvanted vaccine (OVA@Al) could form a long-term antigen storage effect at the injection site by trapping it there and minimizing delivery to draining lymph nodes (LNs) (Fig. 4b). Within 24 h after OVA injection, pure antigen showed a rapid decline at the injection site, which easily entered the lymphatic circulation and quickly spread to the spleen, kidney, liver, and other major organs within 4 h (Fig. 4b, c). As time progressed the antigen distribution in major organs gradually decreased or even cleared. In contrast the OVA@DDAB/PLGA Nv groups showed the excellent ability of efficient antigen delivery to draining LNs. The radioactive signals of proximal LNs as inguinal LNs and popliteal LNs remained strong at 48 h post-injection (Fig. 4b). As time progressed the antigen mainly distributed into the spleen with trace found in the liver, kidneys. What is notable is that even 14 days post-injection antigens were still present in these locations. It was found that the OVA@DDAB/PLGA Nv at the injection site could be transported faster than OVA@Al, but slower than OVA (Fig. 4c). The OVA@DDAB/PLGA Nv not only formed a short-term "antigen repository" for antigen delivery, but also enhanced antigen transportation to LNs. Both of these effects are favorable for inducing an immune response. The Fig. 4d shows the diagram of internal organs in mice.

**Fig. 5:**
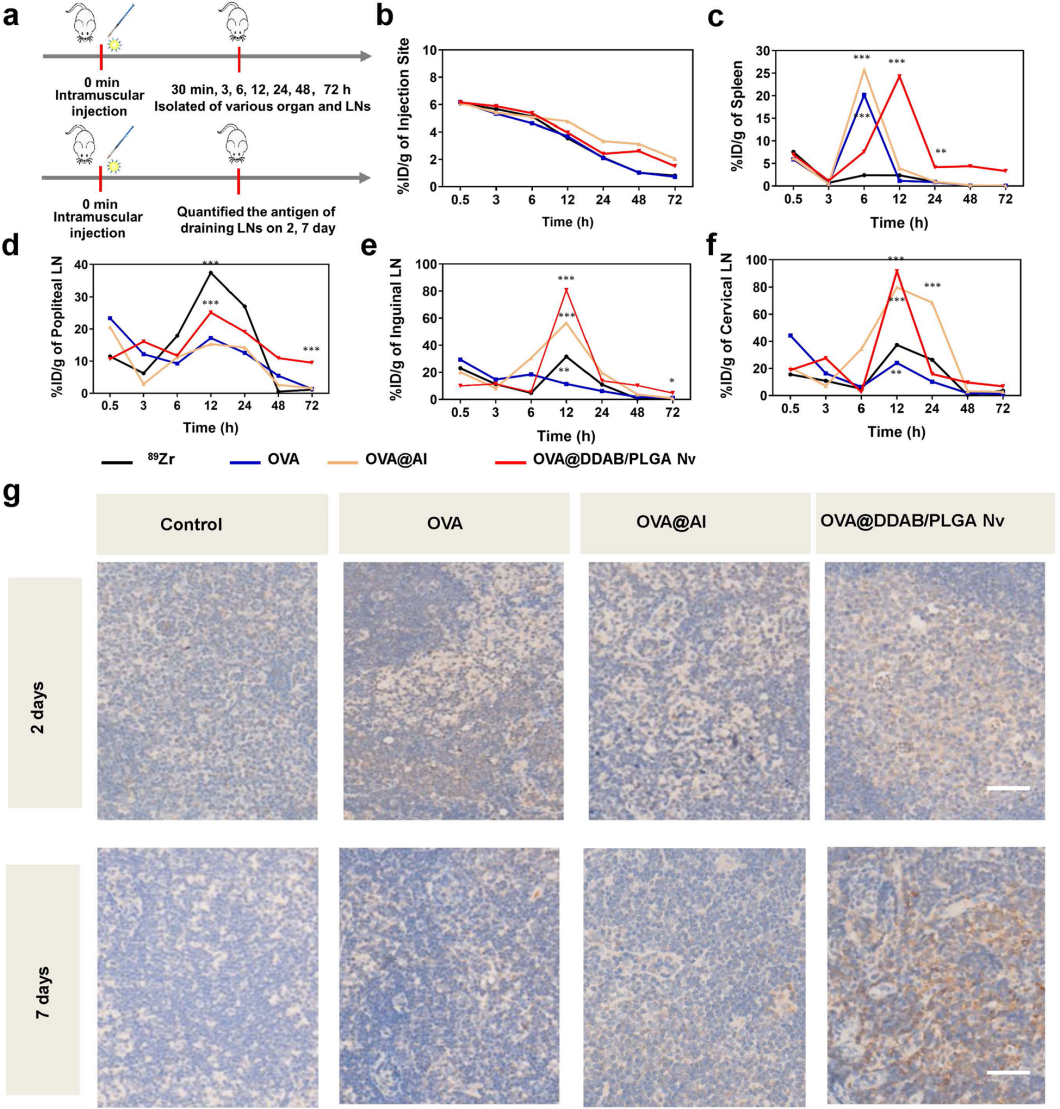
Dynamic antigen biodistribution of different vaccine formulations in mice. **a** Experimental design to evaluate the dynamic antigen biodistribution. **b**–**f** ^89^Zr-OVA biodistribution in injection site (**b**), spleen (**c**) Popliteal LNs (**d**), inguinal LNs (**e**), and cervical LNs (**f**). The primary organs and secondary lymphoid tissues of mice were isolated at 0.5, 3, 6, 12, 24, 48, and 72 h, and then radioactivity was quantified to calculate %ID/g value (% ID/g: antigen uptake rate per gram of tissue at different times). **g** immunohistochemical methods quantified the antigen in draining LNs (the brown areas represent the antigen OVA), and stained sections were measured by the automatic multispectral imaging system (PerkinElmer Vectra II), bar = 200 μm. Data are expressed as Mean ± STD (n = 6)

The biodistribution results indicated that the OVA@DDAB/PLGA Nv could effectively deliver antigen to secondary lymphoid organs such as the spleen and draining lymph nodes. Therefore, we further quantitatively studied the dynamic antigen biodistribution for different formulations in vivo. After intramuscular injection with ^89^Zr-OVA, ^89^Zr-OVA@Al or ^89^Zr-OVA@DDAB/PLGA Nv, the primary and secondary lymphoid tissues of mice were isolated at 0.5, 3, 6, 12, 24, 48, and 72 h to monitor radioactivity for calculating the biodistribution value (Fig. 5a; %ID/g: antigen uptake rate per gram of tissue at different times). The results showed that pure antigen (OVA) could be rapidly transported from the injection site to spleen with antigen uptake peaking at 6 h post injection (Fig. 5b, c). ^89^Zr-OVA@Al showed similar rapid antigen delivery to the spleen and peaking 6 h post injection as well (Fig. 5c). In contrast ^89^Zr-OVA@DDAB/PLGA Nv exhibited delayed antigen delivery to spleen with uptake peaking at 12 h post injection. A notable phenomenon is that ^89^Zr-OVA@DDAB/PLGA Nv showed two antigen transport peaks at 3 h and 12 h in draining LNs while ^89^Zr-OVA and ^89^Zr-OVA@Al only had a single peak at 12 h (Fig. 5d–f). Furthermore, antigen transported to the popliteal and inguinal LNs via the ^89^Zr-OVA@DDAB/PLGA Nv were maintained there for more than 72 h, while the antigen signal from ^89^Zr-OVA and ^89^Zr-OVA@Al dropped down to a very low level after 12 h (Fig. 5d, e). ^89^Zr-OVA@DDAB/PLGA Nv not only enhanced antigen delivery to proximal LNs, but also greatly promoted antigen transport to distal LNs such as cervical LNs (Fig. 5f). According these results, we hypothesize that ^89^Zr-OVA@DDAB/PLGA Nv transported antigen through two pathways: the first was direct transport into draining lymph nodes and the second was indirect transport by immune cells uptake such as DCs.

### OVA@DDAB/PLGA Nv Induced germinal centers formation in draining LNs and proliferative response of splenocyte

Antigen amounts in popliteal and inguinal LNs measured by flow cytometry also confirmed that OVA@DDAB/PLGA Nv promoted much more two folds antigen transport to lymph nodes as popliteal and inguinal LNs at 12 h than OVA and OVA@Al (Additional file 1: Fig. S5; *P* < 0.001). Immunohistochemical images suggested that the OVA@DDAB/PLGA Nv showed much higher ability in transferring antigen to LNs than OVA and OVA@Al (Fig. 5g and Additional file 1: Fig. S6). Moreover, antigens presented with the OVA@DDAB/PLGA Nv persisted in the LNs even seven days post immunization. These results implied that OVA@DDAB/PLGA Nv promoted both antigen migration into draining LNs at the early stage and subsequent continuous antigen stimulation to immune cells in LNs.

Maturation and activation of DCs is a prerequisite of antigen presentation, and directly affects the interaction with T cells [24, 28, 29]. The expression of co-stimulatory molecules CD86 on DCs was determined by flow cytometry. OVA@DDAB/PLGA Nv increased the expression of CD86 molecules on DCs in draining LNs (Additional file 1: Fig. S7a; NVs *vs* pure antigen: p < 0.0001). The expression of MHC I and MHC II on DCs from secondary LNs were also increased, which inducing by OVA@DDAB/PLGA Nv (Additional file 1: Fig. S6b, c). These results indicated that OVA@DDAB/PLGA Nv significantly promoted antigen cross-presentation. Expression of MHC II in the OVA@Al group gradually decreased after 24 h of immunization, while the MHC II in the OVA@DDAB/PLGA Nv group remained high (Additional file 1: Fig. S7c). These higher levels of MHC I and MHC II molecule expression on DCs suggested a stronger MHC-restricted antigen presenting pathway, which was favorable for T cell-mediated immunity. Therefore, these results indicated that the OVA@DDAB/PLGA Nv were superior to OVA@Al for inducing maturation and activation of DCs in secondary lymphocytes.

**Fig. 6:**
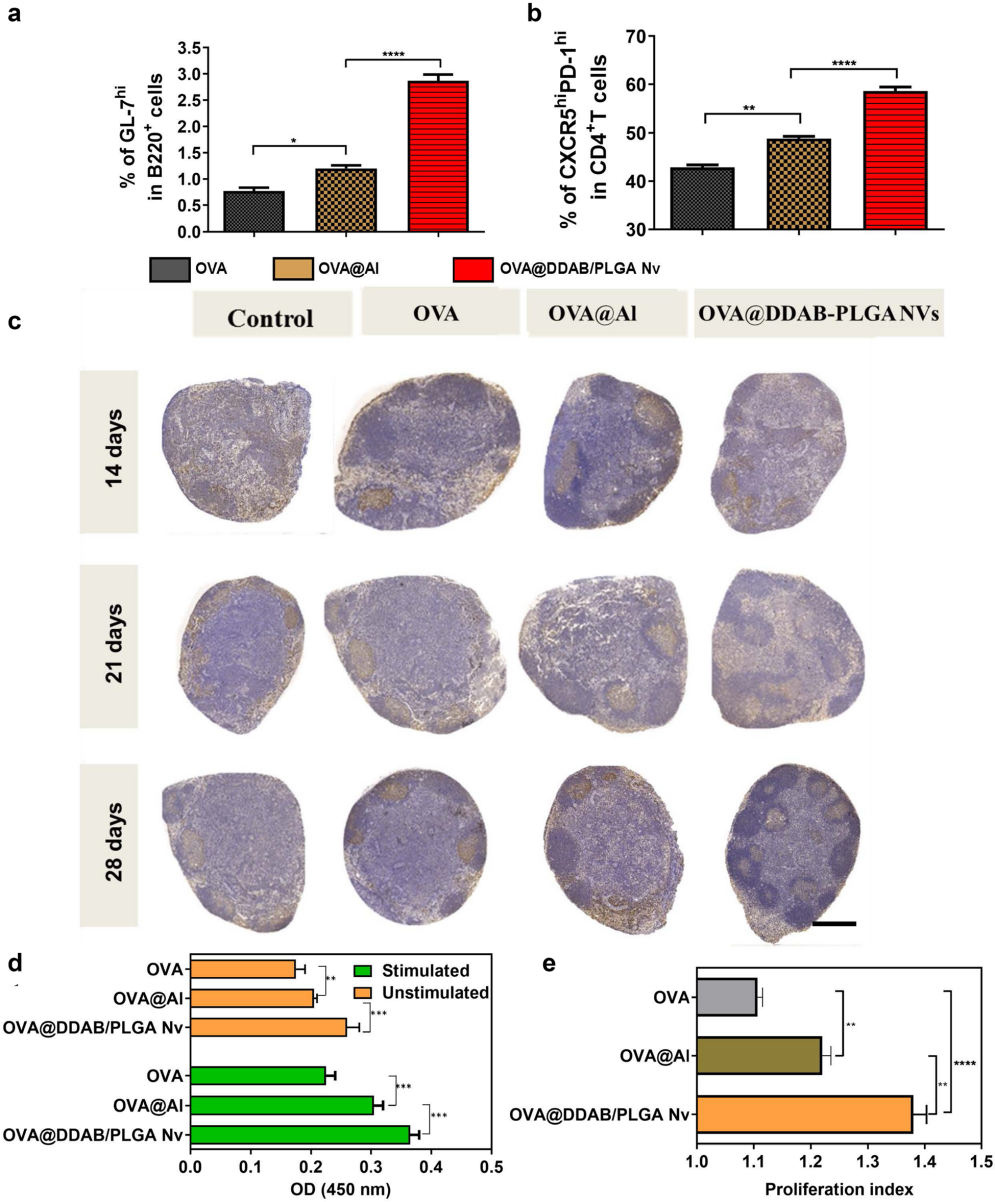
The OVA@DDAB/PLGA Nv induced the formation of germinal centers in draining LNs and proliferative response of splenocytes from immunized mice. **a** The number of germinal center (GL-7^hi^ B220^+^ cells) in draining LNs. **b** The follicular helper CD4^+^ T cells (Tfh, CXCR5^hi^ PD-1^hi^ CD4^+^ T cells) in draining LNs. **c** Germinal centers in draining LNs determined by immunohistochemical staining; The circular brown signal is the germinal center B cell, and the dense circular positive area is the germinal center, which is circular or oval with a diameter of about 0.1 to 1.0 mm, and the bright area can be identified from the outside to the inside, bar = 1.0 mm. **d** OD450 nm value for immunized mice splenocytes stimulated with OVA in vitro were measured by CCK-8 kit; **e** Proliferation index of splenocytes. Data were expressed as means ± SEM (n = 6). (**p < 0.01; ***p < 0.001; ****p < 0.0001)

The OVA@DDAB/PLGA Nv showed great difference in antigen transportation from injection site to LNs and spleen compared to OVA and OVA@Al. Therefore, we further explored whether this difference would have a significant influence on subsequent immune activations. One of the most important functions of vaccines is to induce an effective specific and/or neutralizing antibodies against pathogens, which are mainly related to the level of follicular helper CD4^+^ T cells (Tfh) and formation of germinal centers represent B cells activation capacity. Flow cytometry was employed to detect the populations of follicular helper CD4^+^ T cells (CD4^+^ CXCR5^hi^ PD-l^hi^) and germinal centers (GL-7^hi^ B220^+^) in LNs. The OVA@DDAB/PLGA Nv significantly induced more cells populations of germinal centers in LNs over two-fold than OVA and OVA@Al (Fig. 6a and Additional file 1: Fig. S8b; for OVA and OVA@Al, *P* < 0.0001). And all three groups induced over 40% higher levels of follicular helper CD4^+^ T cells (Fig. 6b and Additional file 1: Fig. S8c). Immunohistochemical results showed that OVA@DDAB/PLGA Nv induced more germinal centers than the other two groups, notably 28 days post immunization (Fig. 6c and S9). Meanwhile, the proliferation and activation of lymphocytes in the spleen was important for immune system stimulation. The splenocyte from mice injected with OVA@DDAB/PLGA Nv showed much stronger proliferation ability than those with OVA (*P* < 0.0001) and OVA-Al (*P* < 0.01) (Fig. 6d-e). These results indicated that the OVA@DDAB/PLGA Nv could effectively promote germinal centers formation and splenocyte proliferation, which would be favorable for an effective immune response.

### OVA@DDAB/PLGA Nv induced the splenocyte activation and cytokines secretion

**Fig. 7:**
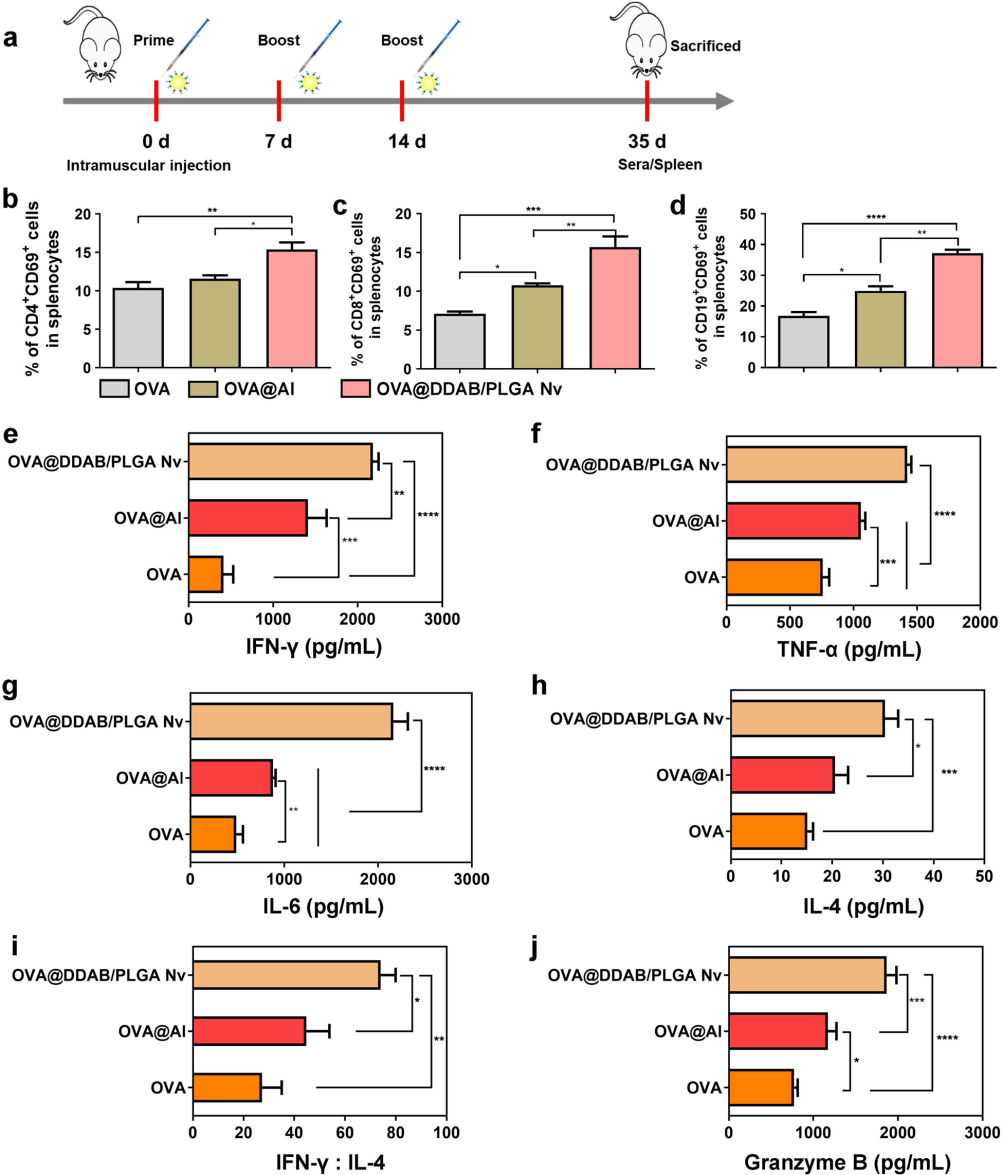
OVA@DDAB/PLGA Nv induced splenocyte activation and cytokines secretion. **a** The schematic treatment schedule of the vaccine immunization. **b**, **c**, **d** The activation of CD4^+^ T cells (**b**) CD8^+^ T cells (**c**) and B cells (**d**) from mice after being immunized with different vaccine formulations were measured by flow cytometry. **e**, **f**, **g**, **h** The secretion of Th1-type cytokine of IFN-γ (**e**), TNF-α (**f**) and Th2-type cytokine of IL-6 (**g**), IL-4 (**h**) after intramuscular injection with different vaccines formulations were measured by an ELISA kit. **i** The ratio of IFN-γ to IL-4 secreted by splenocytes immunized with different vaccines. **j** Granzyme B secretion by splenocytes immunized with different vaccines were measured by an ELISA kit. Data was expressed as Mean ± STD (n = 6). (*p < 0.05; **p < 0.01; ***p < 0.001; ****p < 0.0001)

As a major indicator of lymphocyte activation in the spleen, the antigen specific CD4^+^ T, CD8^+^ T and B cells were evaluated and analyzed by flow cytometry after mice splenocytes were re-stimulated with antigen in vitro. CD69 and CD19 usually serve as the activation marker of T cells and B cells, which was used to measure lymphocyte activation. The mice were immunized three times and the spleen cells were isolated 35 days after the first immunized (Fig. 7a). The immunized splenocytes restimulated with OVA in vitro were employed for measuring CD4^+^ T cells (CD4^+^ CD69^+^ cells), CD8^+^ T cells (CD8^+^ CD69^+^ cells) and B cells (CD19^+^ CD69^+^ cells) by flow cytometry. The results suggested that the OVA@DDAB/PLGA Nv could promote more T and B cells activation than OVA and OVA@Al (Fig. 7b–d and Additional file 1: Fig. S10b–d; for CD4^+^ T cells comparing with OVA, *p* < 0.01; for CD4^+^ T cells comparing with OVA@Al, *p* < 0.05; for CD8^+^ T cells comparing with OVA, *p* < 0.001; for CD8^+^ T cells comparing with OVA@Al, *p* < 0.01; for CD19^+^ B cells comparing with OVA, *p* < 0.0001; for CD19^+^ B cells comparing with OVA@Al, *p* < 0.01). These results indicated that the OVA@DDAB/PLGA Nv could induce a strong activation of effector immune cells (T cells and B cells), which was necessary for enhancing system immune response.

It was reported that cytokines secretion from splenocytes play an essential role in provoking cell-mediated immune response [30]. Th1-type cytokines as IFN-γ, TNF-α and Th2-type cytokines including IL-4, IL-6 were evaluated. It was found that the OVA@DDAB/PLGA Nv significantly promoted more IFN-γ and TNF-α secretion than OVA (*P* < 0.0001) and OVA@Al (*P* < 0.01), and improved IL-6 and IL-4 secretion compared to OVA (*P* < 0.001) and OVA@Al (*P* < 0.05) (Fig. 7e–h). This implies that DDAB/PLGA nanoparticles also functioned as vaccine adjuvants by promoting Th1 and Th2 immune responses. The IFN-γ/IL-4 ratio was an indicator of the Th1/Th2 propensity of the immune response[31]. The ratio of IFN-γ/IL-4 induced by OVA@DDAB/PLGA Nv was higher than OVA (*P* < 0.01) and OVA@Al (*P* < 0.05), which further exhibited that the OVA@DDAB/PLGA Nv were more prone to induce Th1 type immune response (Fig. 7i). Meanwhile, the Nv induced higher levels of Granzyme B (Fig. 7j). The Granzyme B is an exogenous serine protease, which is derived from cytoplasm particles released by CTLs and natural killer cells (NK). Granzyme B plays an important role in cellular immune response, which can induce DNA degradation of target cells as infected cells and then lysis by activating the chain reaction of caspases. Therefore, our analysis indicated that the OVA@DDAB/PLGA Nv increased Th1 type cytokines secretion, which regulated the activation of immune cells and further induced the production of high levels of Granzyme B. In summary, the OVA@DDAB/PLGA Nv could effectively deliver antigens to LNs and spleen to induce a stronger immune response than OVA and OVA@Al, which demonstrated that the OVA@DDAB/PLGA Nv could induced not only cellular immunity from Th1 and Th2 responses, but also humoral immunity from B cells.

### OVA@DDAB/PLGA Nv promoted T-cell-mediated immune response and elicited superior antibody response in mice

**Fig. 8:**
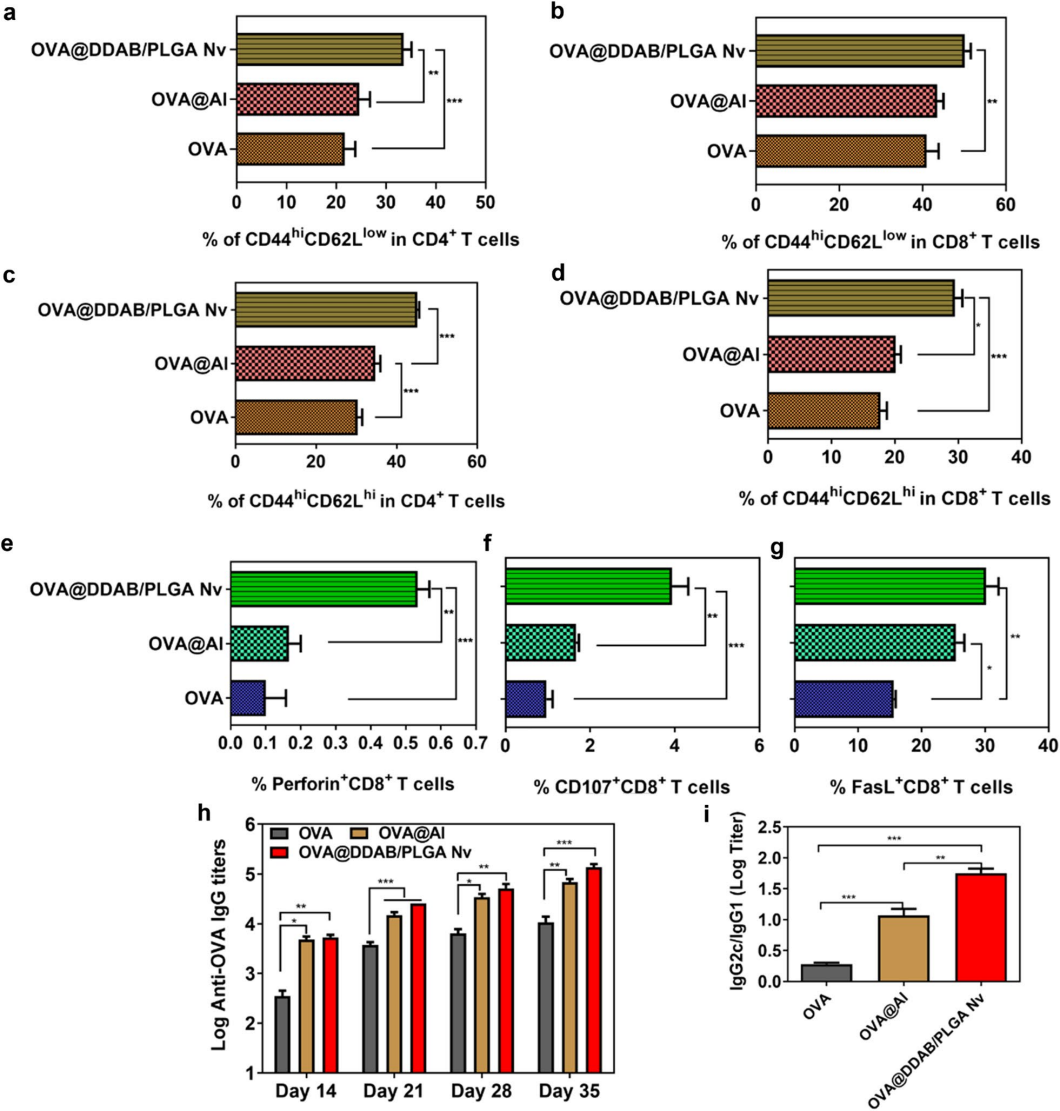
Effects of different vaccines on memory T cell responses, CTL response, and antibody levels. **a** Effector memory (CD44^hi^ CD62L^low^) CD4^+^ T cells, **b** Cells of effector memory (CD44^hi^ CD62L^low^) CD8^+^ T cells, **c** Central memory (CD44^hi^ CD62L^hi^) CD4^+^ T cells, and **D** Central memory (CD44^hi^ CD62L^hi^) CD8^+^ T cells. The expression of Perforin (**e**), CD107 (**f**), and FasL (**g**) on CD8^+^ T cell in splenocytes. **h** Anti-OVA IgG titers in the serum from mice after being immunized with different vaccines for 35 days. **i** The composition ratio of IgG1 and IgG2c in serum from mice after immunized with different vaccines for 35 days. Data were analyzed by flow cytometry and expressed as Mean ± STD (n = 6). (*p < 0.05; **p < 0.01; ***p < 0.001)

The ultimate goal of vaccination is to generate immune memory that can rapidly respond to pathogens upon reinfection, and memory T cells are an important components of these memory immune responses [32]. CD44^hi^ CD62L^low^ and CD44^hi^ CD62L^hi^ are regarded as markers for effector-memory T cells and central-memory T cells, respectively [32–34]. In our study, the OVA@DDAB/PLGA Nv generated significantly more effector T cells of CD4^+^ T and CD8^+^ T cells than OVA and OVA@Al (Fig. 8a, b and Additional file 1: Fig. S11b). Meanwhile, the OVA@DDAB/PLGA Nv also increased central-memory T cells of CD4^+^ T and CD8^+^ T cells which in theory will ensure a rapid immune response that will protect against reinfection (Fig. 8c, d and Additional file 1: Fig. S11b). In addition, cytotoxic T lymphocytes (CTL), a specific type of T cell, secretes a variety of cytokines and participates in the immune response. Its most important function is the specific killing effect on the pathogens that cause cellular infection [35]. CTL activation promotes the expression of CD107 molecules on the surface of CD8^+^ T cells [36]. Studies have found that CTL exerts its anti-killing effect mainly through two mechanisms: the first is the release of perforin and granzyme to kill target cells and the second is FasL-mediated cell apoptosis [37]. Analysis of the expression of CTL-related molecules by flow cytometry was an important indicator for evaluating immune response induced by vaccines. Meanwhile, the OVA@DDAB/PLGA Nv increased perforin release from CD8^+^ T cells compared to OVA (Fig. 8e and Additional file 1: Fig. S12b; *P* < 0.001). Compared to OVA, the OVA@DDAB/PLGA Nv could significantly up-regulate the CD107 and FasL expression on CD8^+^ T cells (Fig. 8f-g and Additional file 1: Fig. S12c, d; *P* < 0.01). These indicated that the OVA@DDAB/PLGA Nv could promote CTL activation and enhance CD107/FasL/perforin-mediated immune killing. In combination with the increasing expression of granzymes, we confirmed that the OVA@DDAB/PLGA Nv mainly relied on the mechanisms of Granzyme B and CD107/FasL/perforin expression to induce CTL responses.

As the main immunoglobulin, Immunoglobulin G (IgG) is a key index to evaluate the level of immune response [38]. IgG1 and IgG2 are the two main subtypes of IgG. IgG2c/IgG1 can reflect the Th1/Th2 tendency in the immune response [32, 39, 40]. In this study, specific antibody titers in serum were detected by ELISA. We measured the antigen-specific IgG, IgG1, and IgG2c titers in serum from immunized mice with different vaccines. The results showed that OVA-specific antibody IgG gradually increased on day 14, 21, 28 and 35, and the IgG titers from OVA@Al and OVA@DDAB/PLGA Nv were much higher than OVA (*P* < 0.01), indicating that both mice groups exhibited stronger anti-OVA IgG responses (Fig. 8h). The OVA@DDAB/PLGA Nv induced higher IgG levels, while it induced stronger humoral immune response than OVA (for Day 21 and Day 35, *P* < 0.001; for Day 14 and Day 28, *P* < 0.01) (Fig. 8h), which suggested that the OVA@DDAB/PLGA Nv could induced humoral immunity. In addition, OVA@DDAB/PLGA Nv significantly increased much higher IgG2c proportion in the total IgG than OVA (*P* < 0.001) and OVA@Al (*P* < 0.01) (Fig. 8i), which indicated that the OVA@DDAB/PLGA Nv was capable of enhancing Th1 immune response (cellular immune response).

In addition to the immune effects, we also evaluated the safety of our OVA@DDAB/PLGA Nv delivery system. Blood samples were collected for the biochemistry of urea nitrogen (BUN), aspartate transaminase (AST), alanine aminotransferase (ALT), alkaline phosphatase (ALP), and lactate dehydrogenase (LDH). Among these parameters, the values of BUN, AST and LDH were withing normal ranges for the OVA@DDAB/PLGA Nv group (Additional file 1: Fig. S13a, b, e). In contrast, ALT and ALP were found to be abnormally enhanced by Al adjuvant (Additional file 1: Fig. S13c-d), suggesting that this treatment had effectively spared the mice from hepatic or other organ damage. This indicates that its safer using DDAB/PLGA NP than Al adjuvant for immune protection and therapy.

## Conclusion

In this paper, we successfully prepared OVA-loaded DDAB-PLGA nanoparticles (OVA@DDAB/PLGA Nv) with a positive charge. We explored the signal pathways that induced DCs activation and maturation in vitro and stimulated immune responses in vivo by the OVA@DDAB/PLGA Nv. Experiments performed in vitro show that the OVA@DDAB/PLGA Nv promoted the cellular uptake of antigen and the activation of BMDCs. The antigen uptake capacity of BMDCs incubated with OVA@DDAB/PLGA Nv was improved by more than twofold relative to pure OVA and OVA@Al at 12 h. Moreover, OVA@DDAB/PLGA Nv promoted antigen escape from the lysosomes into the cytoplasm, and induced ten times higher antigen cross-presentation activity compared to those OVA and OVA@Al. It was found that OVA@DDAB/PLGA Nv and OVA@Al exhibited different pathways to activate immunity. OVA@DDAB/PLGA Nv mainly activated DCs by p38 MAPK signal pathway. Meanwhile OVA@Al did not completely rely on the p38 signal pathway. Moreover, OVA@DDAB/PLGA Nv demonstrated two antigen transport pathways: one was direct rapid transport into draining lymph nodes at 3 h, and the other was an indirect transport pathway by DCs uptake at 12 h. These transport routes ensured that the OVA@DDAB/PLGA Nv could rapidly and continuously deliver antigen to the lymph nodes, which is favorable for stimulating an immune response in vivo. The antigen^+^ cells in inguinal LNs and popliteal LNs for OVA@DDAB/PLGA Nv were increased more than threefold compared to those of OVA@Al and OVA at 24 h. Furthermore, the antigen of OVA@DDAB/PLGA Nv persisted in LNs for more than 7 days, which induced germinal center formation as two times higher than those of OVA@Al and OVA. After immunization, OVA@DDAB/PLGA Nv induced comparable anti-OVA IgG and much higher ratio of IgG2c/IgG1 compared to OVA@Al. It also effectively activated CD4^+^T, CD8^+^T and B cells that further induced immune memory. As expected, OVA@DDAB/PLGA Nv elicited a strong cytotoxic T lymphocytes (CTLs) response. Stimulation with OVA@DDAB/PLGA Nv increased the proportion of CD107^+^CD8^+^, Fasl^+^CD8^+^ and perforin^+^CD8^+^ T cells and the secretion of IFN-γ and Granzyme B. These results indicate that the DDAB/PLGA NP was a potent platform to improve vaccine immunogenicity by novel signaling pathway in DCs, rapid and massive transport antigens to LNs.

## Supplementary Information

**Additional file 1: Table S1.** Characterization of PLGA nanoparticles (PLGA NPs), DDAB/PLGA NPs, and DDAB/PLGA Nano-vaccines (OVA@DDAB/PLGA Nv) (Mean ± STD).** Figure S1.** Cytotoxicity of OVA@DDAB/PLGA Nv.** Figure S2.** Expression of co-stimulatory molecules from DCs stimulated with different formulations. (a) the gating strategies of flow cytometry of activated DC cells. (b-d) Percentages of CD40+ CD11c+ (b), CD86+ CD11c+ (c) and MHC II+ CD11c+ (d) cells were analyzed by flow cytometry.** Figure S3.** (a) The changes of p38 MAPK, p-AKT and p-ERK phosphorylation after being stimulated with different formulation DCs for 6 h and 12 h. (b) Change of the p38 MAPK, p-AKT and p-ERK phosphorylation level by stimulated DCs with different concentration of DDAB-PLGA Nv. (c) DDAB-PLGA Nv increased the binding of MKK3 to its substrate p38α.** Figure S4.** The radiochemical purity of [89Zr]-Df-Bz-NCS-OVA incubated at room temperature, saline (37℃) and fresh serum (37℃) over the course of 14 days.** Figure S5.** The proportion of antigen-carrying cells in different LNs as analyzed by flow cytometry. Three mice were analyzed in every group (n = 3), and data are the mean ± SEM and representative of three independent experiments. Differences between two groups were tested using an unpaired, two-tailed Student’s t-test. Differences among multiple groups were tested with one-way ANOVA followed by Tukey’s multiple comparison. Significant differences between groups are expressed as follows: *P < 0.05, **P < 0.01, or ***P < 0.001.** Figure S6.** The proportion of antigen-carrying cells in different LNs as analyzed by immumohistochemical staining. The data were analyzed by automatic multispectral imaging system (PerkinElmer Vectra II). Three mice were analyzed in every group (n = 3), and data are the mean ± SEM and representative of three independent experiments. Differences between two groups were tested using an unpaired, two-tailed Student’s t-test. Differences among multiple groups were tested with one-way ANOVA followed by Tukey’s multiple comparison. Significant differences between groups are expressed as follows: *P < 0.05, **P < 0.01, or ***P < 0.001.** Figure S7.** Activation and maturation of BMDCs in LNs in vivo. (a–c) Expression of activation markers (CD86, MHC I and MHC II) of DCs in draining LNs.Three mice were analyzed in every group (n = 3), and data are the mean ± SEM and representative of three independent experiments. Differences between two groups were tested using an unpaired, two-tailed Student’s t-test. Differences among multiple groups were tested with one-way ANOVA followed by Tukey’s multiple comparison. Significant differences between groups are expressed as follows: *P < 0.05, **P < 0.01, or ***P < 0.001.** Figure S8.** The OVA@DDAB/PLGA Nv induced the formation of germinal centers in draining LNs. (a) The gating strategies of flow cytometry of germinal center and follicular helper CD4+ T cells. (b) The count of germinal center (GL-7hi B220+ cells) and (c) the follicular helper CD4+ T cells (Tfh, CXCR5hi PD-1hi CD4+ T cells) in draining LNs were analyzed by flow cytometry.** Figure S9.** Germinal centers in draining LNs determined by immunohistochemical staining. The data were analyzed by automatic multispectral imaging system (PerkinElmer Vectra II). Three mice were analyzed in every group (n = 3), and data are the mean ± SEM and representative of three independent experiments. Differences between two groups were tested using an unpaired, two-tailed Student’s t-test. Differences among multiple groups were tested with one-way ANOVA followed by Tukey’s multiple comparison. Significant differences between groups are expressed as follows: *P < 0.05, **P < 0.01, or ***P < 0.001.** Figure S10.** OVA@DDAB/PLGA Nv induced splenocyte activation. (a) The gating strategies of flow cytometry of splenocyte activation. (b, c, d) The activation of CD4+ T cells (b) CD8+ T cells (c) and B cells (d) from mice after being immunized with different vaccine formulations were measured by flow cytometry.** Figure S11.** Effects of different vaccines on memory T cell responses. (a) The gating strategies of flow cytometry of memory T cell. (b) Effector memory (CD44hi CD62Llow) andcentral memory (CD44hi CD62Lhi) in CD4+ and CD8+ T cells were measured by flow cytometry.** Figure S12.** Effects of different vaccines on CTL response. (a) The gating strategies of flow cytometry of CTL cells. The expression of Perforin (b), CD107 (c), and FasL (d) on CD8+ T cell in splenocytes were measured by flow cytometry.** Figure S13.** In vivo toxicity evaluation of DDAB-PLGA Nv. Hematological analysis of treated mice after 35 days. The range marked by dotted lines represents the normal range of different biosafety indicators. The determination of serum biochemistry of urea nitrogen (BUN) (a), aspartate transaminase (AST) (b), alanine aminotransferase (ALT) (c), alkaline phosphatase (ALP) (d), and lactate dehydrogenase (LDH). Three mice were analyzed in every group (n = 3), and data are the mean ± SEM and representative of three independent experiments. Differences between two groups were tested using an unpaired, two-tailed Student’s t-test. Differences among multiple groups were tested with one-way ANOVA followed by Tukey’s multiple comparison. Significant differences between groups are expressed as follows: *P < 0.05, **P < 0.01, or ***P < 0.001.
