# Supplementary material for: Intracellular signaling pathway in dendritic cells and antigen transport pathway in vivo mediated by an OVA@DDAB/PLGA nano-vaccine
Source: J Nanobiotechnology. 2021 Nov 27;19:394. doi: 10.1186/s12951-021-01116-8 (PMC8626881; doi:10.1186/s12951-021-01116-8)
Supplement: Supplementary file 1 — Additional file 1: Table S1. Characterization of PLGA nanoparticles (PLGA NPs), DDAB/PLGA NPs, and DDAB/PLGA Nano-vaccines (OVA@DDAB/PLGA Nv) (Mean ± STD). Figure S1. Cytotoxicity of OVA@DDAB/PLGA Nv. Figure S2. Expression of co-stimulatory molecules from DCs stimulated with different formulations. (a) the gating strategies of flow cytometry of activated DC cells. (b-d) Percentages of CD40+ CD11c+ (b), CD86+ CD11c+ (c) and MHC II+ CD11c+ (d) cells were analyzed by flow cytometry. Figure S3. (a) The changes of p38 MAPK, p-AKT and p-ERK phosphorylation after being stimulated with different formulation DCs for 6 h and 12 h. (b) Change of the p38 MAPK, p-AKT and p-ERK phosphorylation level by stimulated DCs with different concentration of DDAB-PLGA Nv. (c) DDAB-PLGA Nv increased the binding of MKK3 to its substrate p38α. Figure S4. The radiochemical purity of [89Zr]-Df-Bz-NCS-OVA incubated at room temperature, saline (37℃) and fresh serum (37℃) over the course of 14 days. Figure S5. The proportion of antigen-carrying cells in different LNs as analyzed by flow cytometry. Three mice were analyzed in every group (n = 3), and data are the mean ± SEM and representative of three independent experiments. Differences between two groups were tested using an unpaired, two-tailed Student’s t-test. Differences among multiple groups were tested with one-way ANOVA followed by Tukey’s multiple comparison. Significant differences between groups are expressed as follows: *P < 0.05, **P < 0.01, or ***P < 0.001. Figure S6. The proportion of antigen-carrying cells in different LNs as analyzed by immumohistochemical staining. The data were analyzed by automatic multispectral imaging system (PerkinElmer Vectra II). Three mice were analyzed in every group (n = 3), and data are the mean ± SEM and representative of three independent experiments. Differences between two groups were tested using an unpaired, two-tailed Student’s t-test. Differences among multiple groups were tested with one-way A [file 12951_2021_1116_MOESM1_ESM.docx]

**Intracellular Signaling Pathway in Dendritic Cells and Antigen Transport Pathway *In vivo* Mediated by** **an OVA@DDAB/PLGA Nano-vaccine**

Shulan Han^1, 2, 3†^, Wenyan Ma^1, 2, 4†^, Dawei Jiang^5†^, Logan Sutherlin^6^, Jing Zhang^1,2^, Yu Lu^7^, Nan Huo^8^, Zhao Chen^9^, Jonathan W. Engle^6^, Yanping Wang^4*^, Xiaojie Xu^8*^, Lei Kang^9*^, Weibo Cai^6*^, Lianyan Wang^1,2*^

1. *Key Laboratory of Green Process and Engineering, State Key Laboratory of Biochemical Engineering, Institute of Process Engineering, Chinese Academy of Sciences, Beijing 100190, P.R. China.*
2. *University of Chinese Academy of Sciences, Beijing 100049, P.R. China*
3. *School of Pharmaceutical Sciences, Jilin University, Changchun 130021, P.R. China*
4. *Tianjin University of Science and Technology, Tianjin 300222, P.R. China*
5. *Department of Nuclear Medicine, Union Hospital, Tongji Medical College, Huazhong University of Science and Technology, Wuhan 430022, P.R. China.*
6. *Departments of Radiology and Medical Physics, University of Wisconsin - Madison, Madison, Wisconsin 53705, United States*
7. *Institute of Veterinary Immunology &Engineering, Jiangsu Academy of Agricultural Sciences, Nanjing 210014, P.R. China.*
8. *Department of Genetic Engineering Laboratory,* *Beijing Institute of Biotechnology, Beijing 100850, P.R. China.*
9. *Department of Nuclear Medicine, Peking University First Hospital, Beijing 100034, P.R. China*

*†: These authors contributed equally to this work.*

***: *Corresponding author.*

*Address correspondence to:*

[*ypwang@tust.edu.cn*](mailto:ypwang@tust.edu.cn) *(Y.-P. Wang)*

[*wcai@uwhealth.org*](mailto:wcai@uwhealth.org) *(W. Cai)*

[*kanglei@bjmu.edu.cn*](mailto:kanglei@bjmu.edu.cn) *(L. Kang)*

[*miraclexxj@126.com*](mailto:miraclexxj@126.com) *(X.-J. Xu)*

*[wanglianyan@ipe.ac.cn](mailto:wanglianyan@ipe.ac.cn) (L.-Y. Wang)*

**Supporting information**

Table S1. Characterization of PLGA nanoparticles. The size,PDI and zeta of PLGA NPs, DDAB/PLGA NPs, and DDAB/PLGA Nano-vaccines (OVA@DDAB/PLGA Nv) (Mean ± STD) are shown in tableS1.


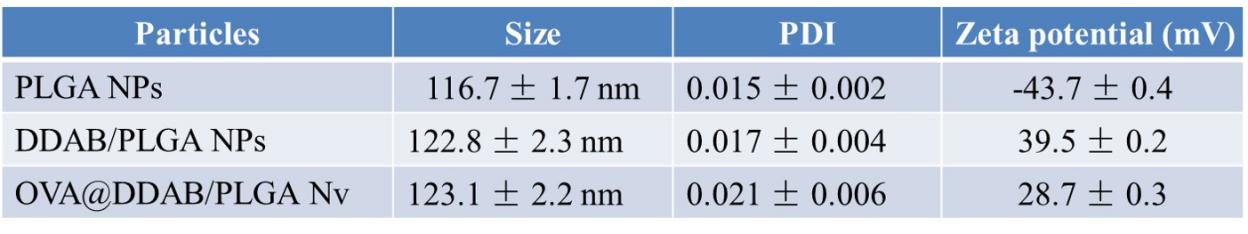





Figure S1. Cytotoxicity of OVA@DDAB/PLGA Nv.


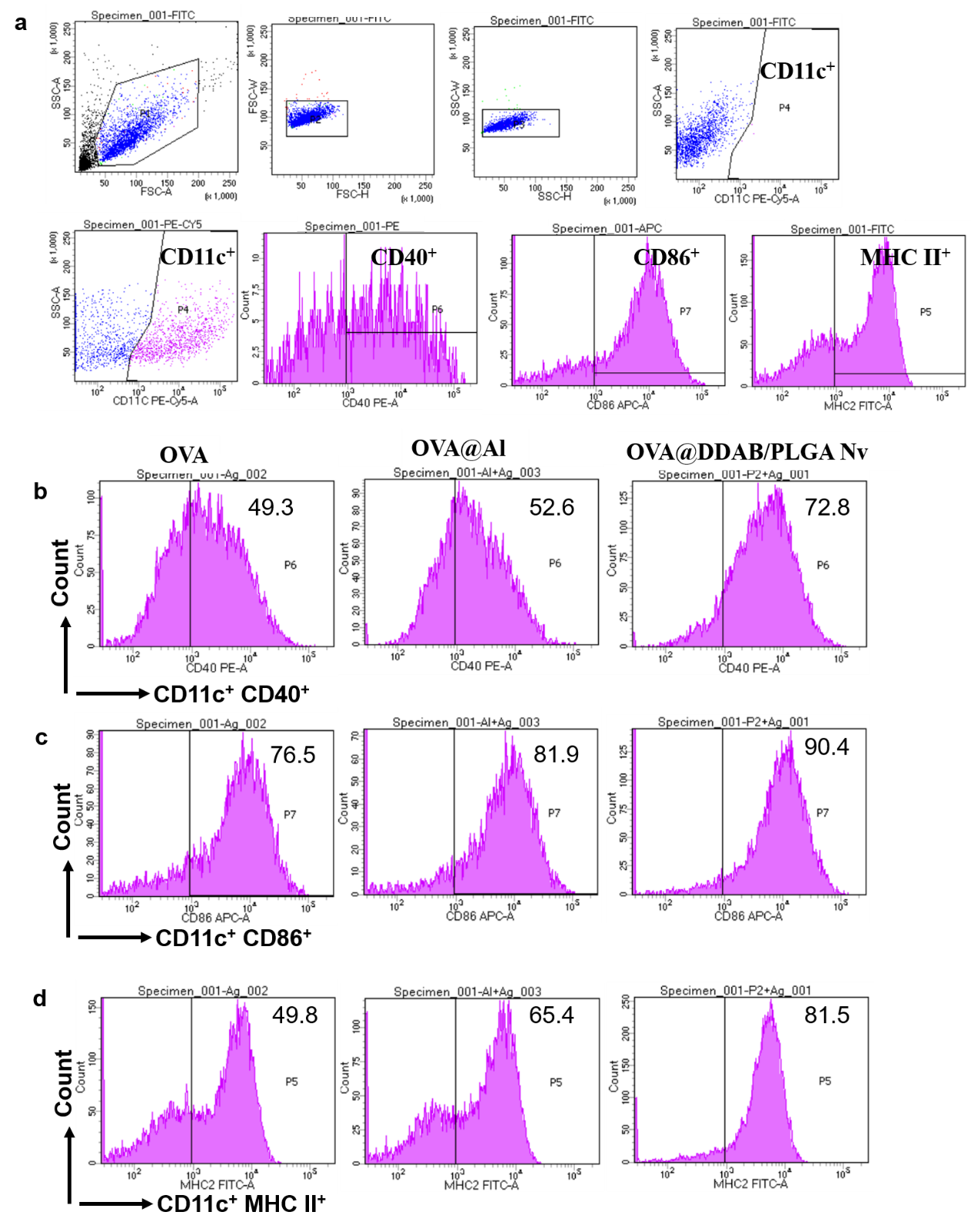


Figure S2. Expression of co-stimulatory molecules from DCs stimulated with different formulations. (a) the gating strategies of flow cytometry of activated DC cells. (b-d) Percentages of CD40^+^ CD11c^+^ (b), CD86^+^ CD11c^+^ (c) and MHC II^+^ CD11c^+^ (d) cells were analyzed by flow cytometry.


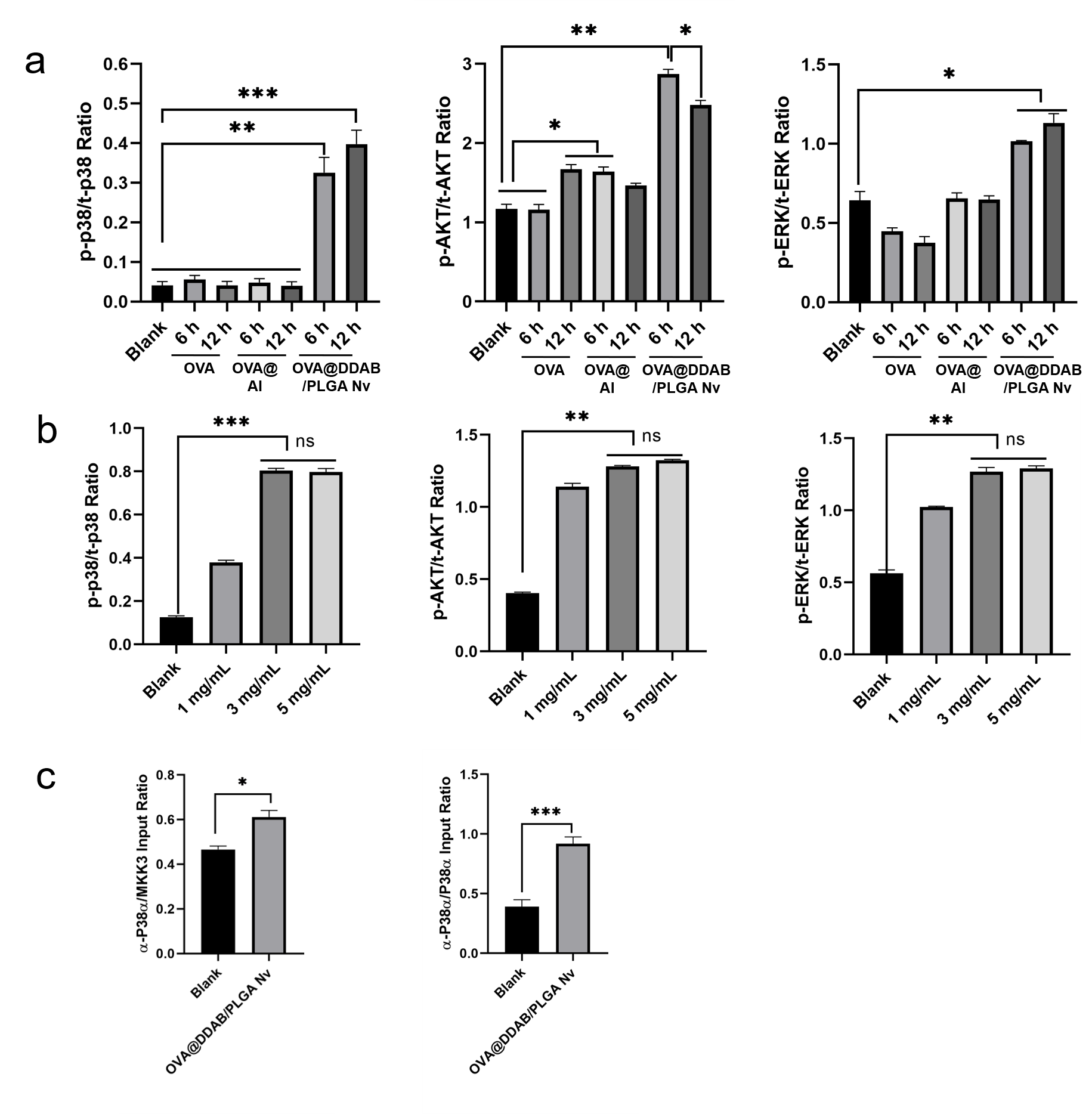


Figure S3.Qualification of protein bands in Fig. 3d, 3e and 3f. (a) The changes of p38 MAPK, p-AKT and p-ERK phosphorylation after being stimulated with different formulation DCs for 6 h and 12 h. (b) Change of the p38 MAPK, p-AKT and p-ERK phosphorylation level by stimulated DCs with different concentration of DDAB-PLGA Nv. (c) DDAB-PLGA Nv increased the binding of MKK3 to its substrate p38α.


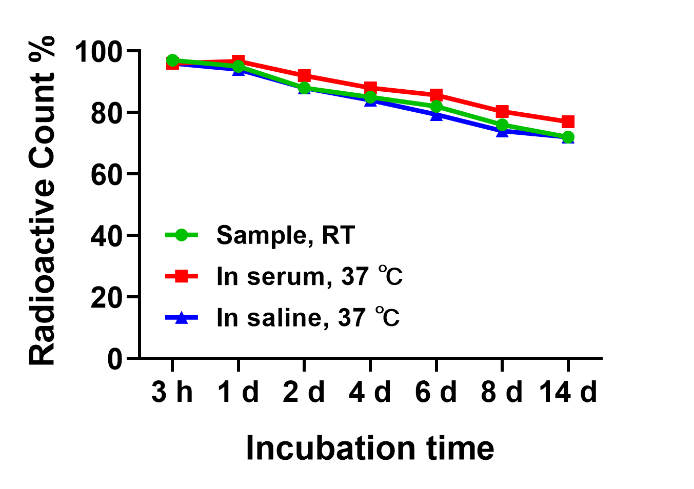


Figure S4. The stability of [^89^Zr]-Df-Bz-NCS-OVA. The radiochemical purity of [^89^Zr]-Df-Bz-NCS-OVA incubated at room temperature, saline (37℃) and fresh serum (37℃) over the course of 14 days.


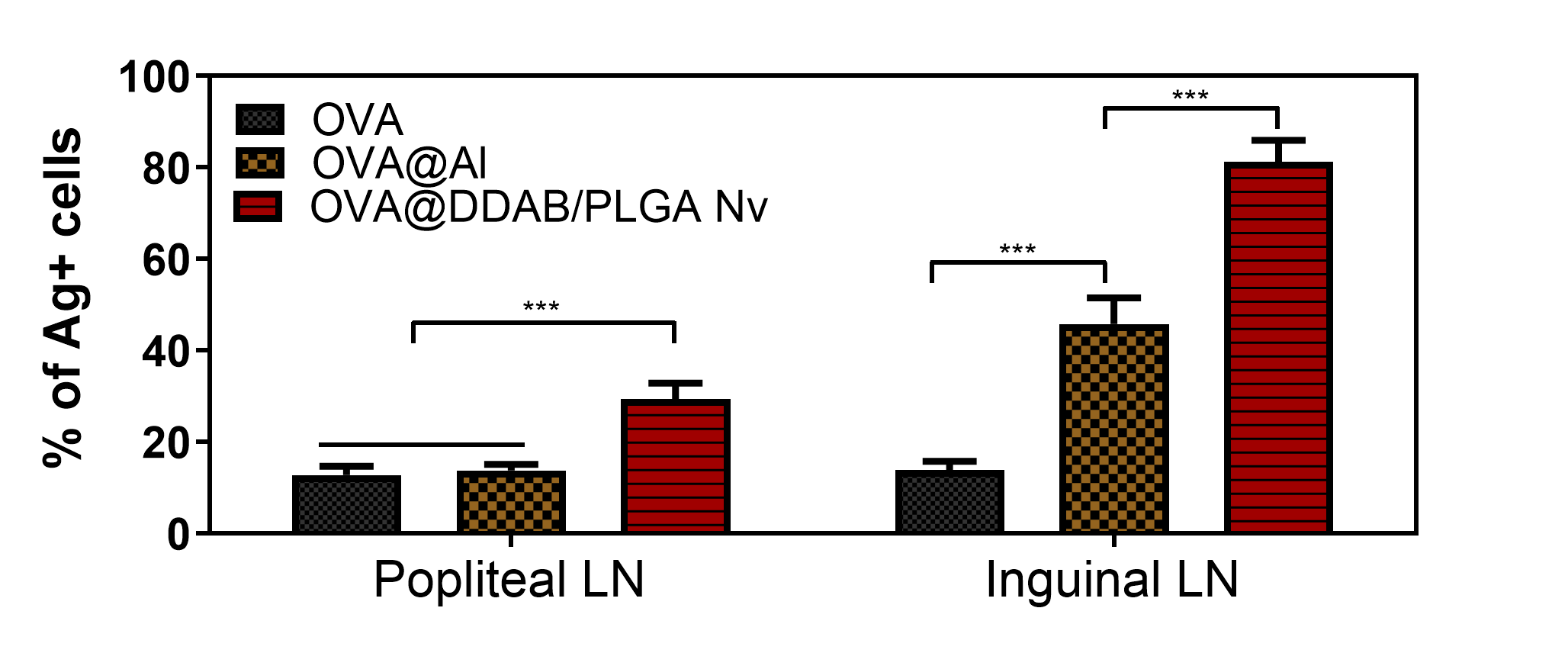


Figure S5. The proportion of antigen-carrying cells in different LNs as analyzed by flow cytometry. Three mice were analyzed in every group (n = 3), and data are the mean ± STD and representative of three independent experiments. Differences between two groups were tested using an unpaired, two-tailed Student’s t-test. Differences among multiple groups were tested with one-way ANOVA followed by Tukey’s multiple comparison. Significant differences between groups are expressed as follows: *P < 0.05, **P < 0.01, or ***P < 0.001.


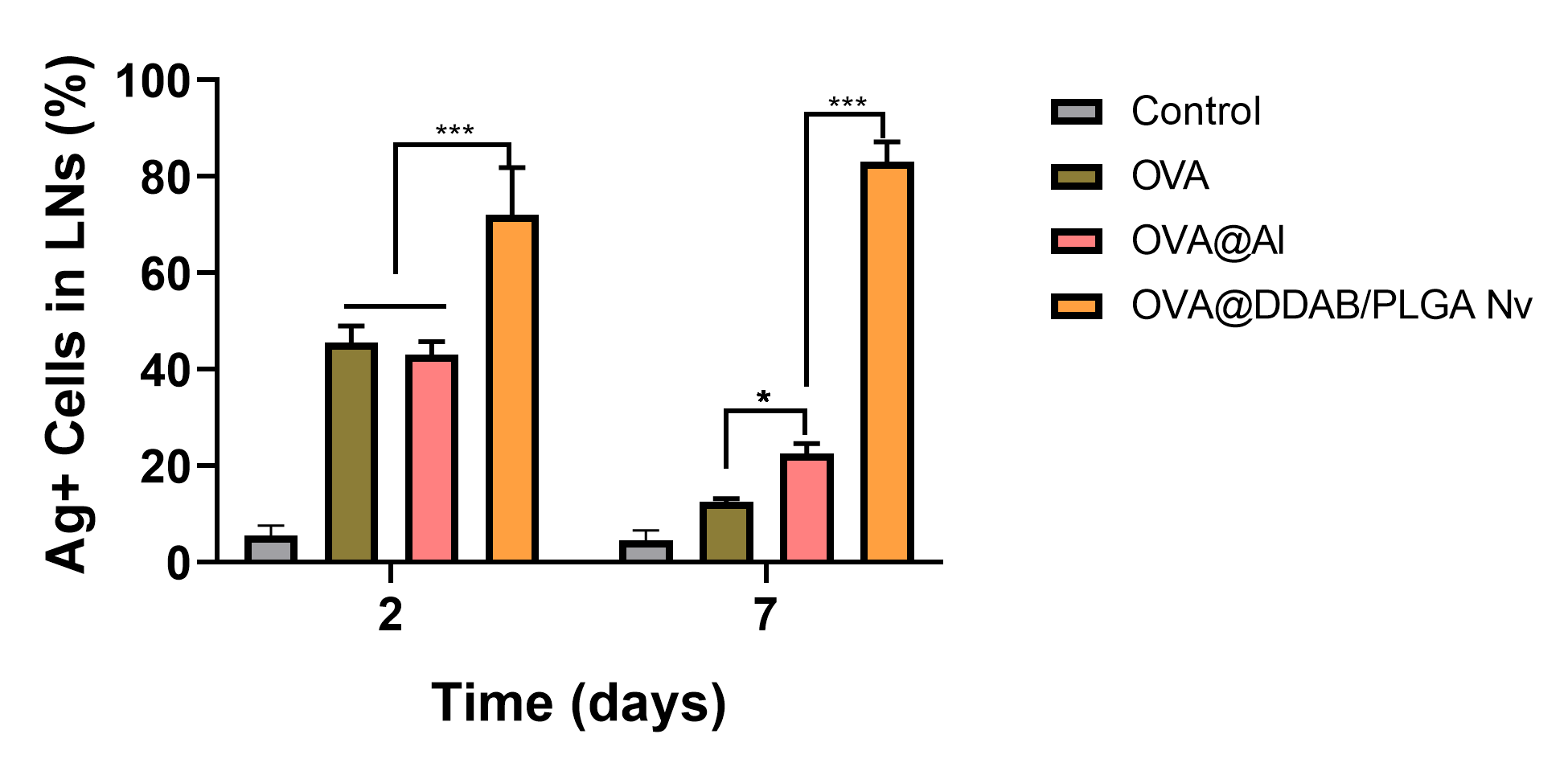


Figure S6. The proportion of antigen-carrying cells in different LNs as analyzed by immumohistochemical staining. The data were analyzed by automatic multispectral imaging system (PerkinElmer Vectra II). Three mice were analyzed in every group (n = 3), and data are the mean ± STD and representative of three independent experiments. Differences between two groups were tested using an unpaired, two-tailed Student’s t-test. Differences among multiple groups were tested with one-way ANOVA followed by Tukey’s multiple comparison. Significant differences between groups are expressed as follows: *P < 0.05, **P < 0.01, or ***P < 0.001.


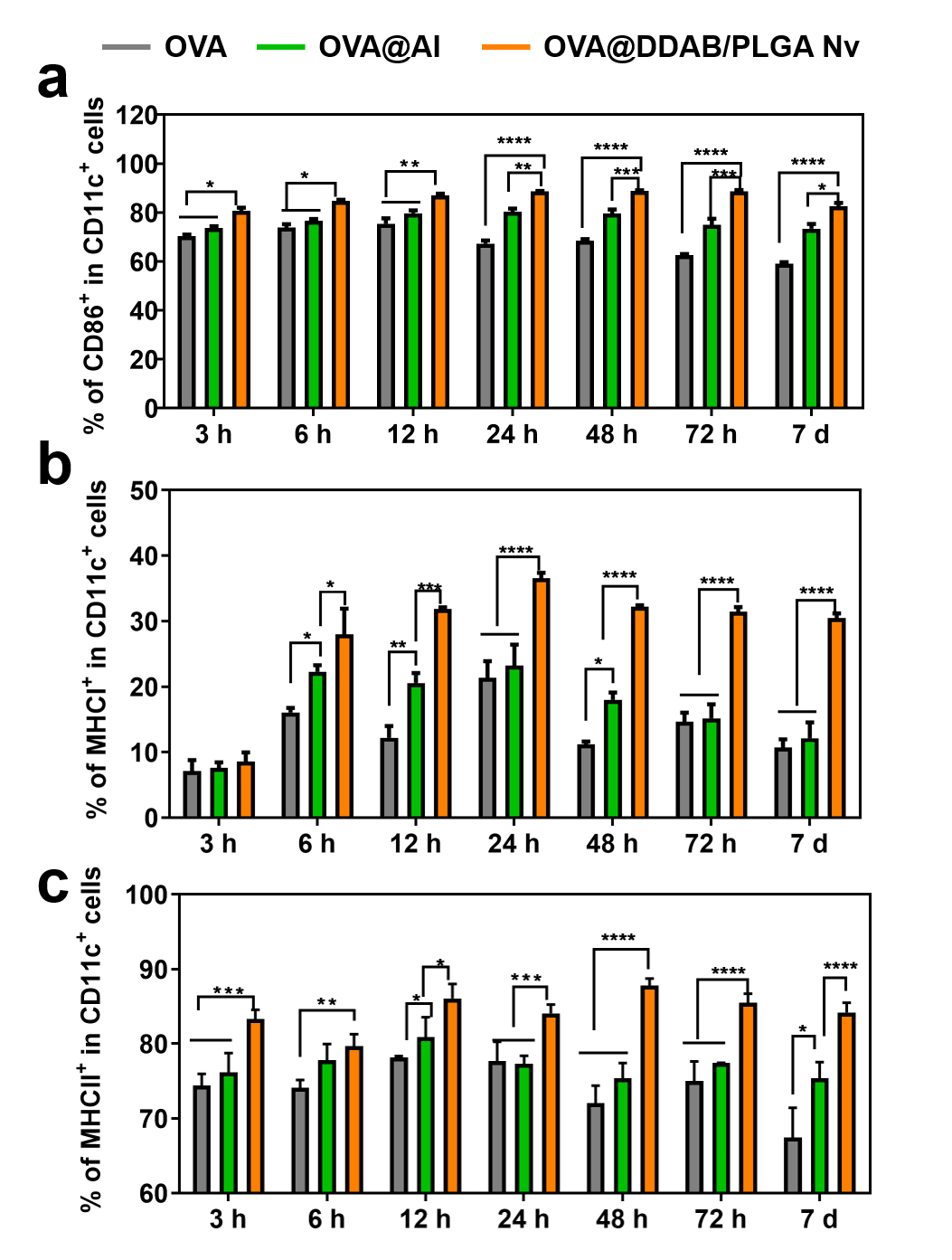


Figure S7. Activation and maturation of BMDCs in LNs *in vivo*. (a-c) Expression of activation markers (CD86, MHC I and MHC II) of DCs in draining LNs.Three mice were analyzed in every group (n = 3), and data are the mean ± STD and representative of three independent experiments. Differences between two groups were tested using an unpaired, two-tailed Student’s t-test. Differences among multiple groups were tested with one-way ANOVA followed by Tukey’s multiple comparison. Significant differences between groups are expressed as follows: *P < 0.05, **P < 0.01, or ***P < 0.001.


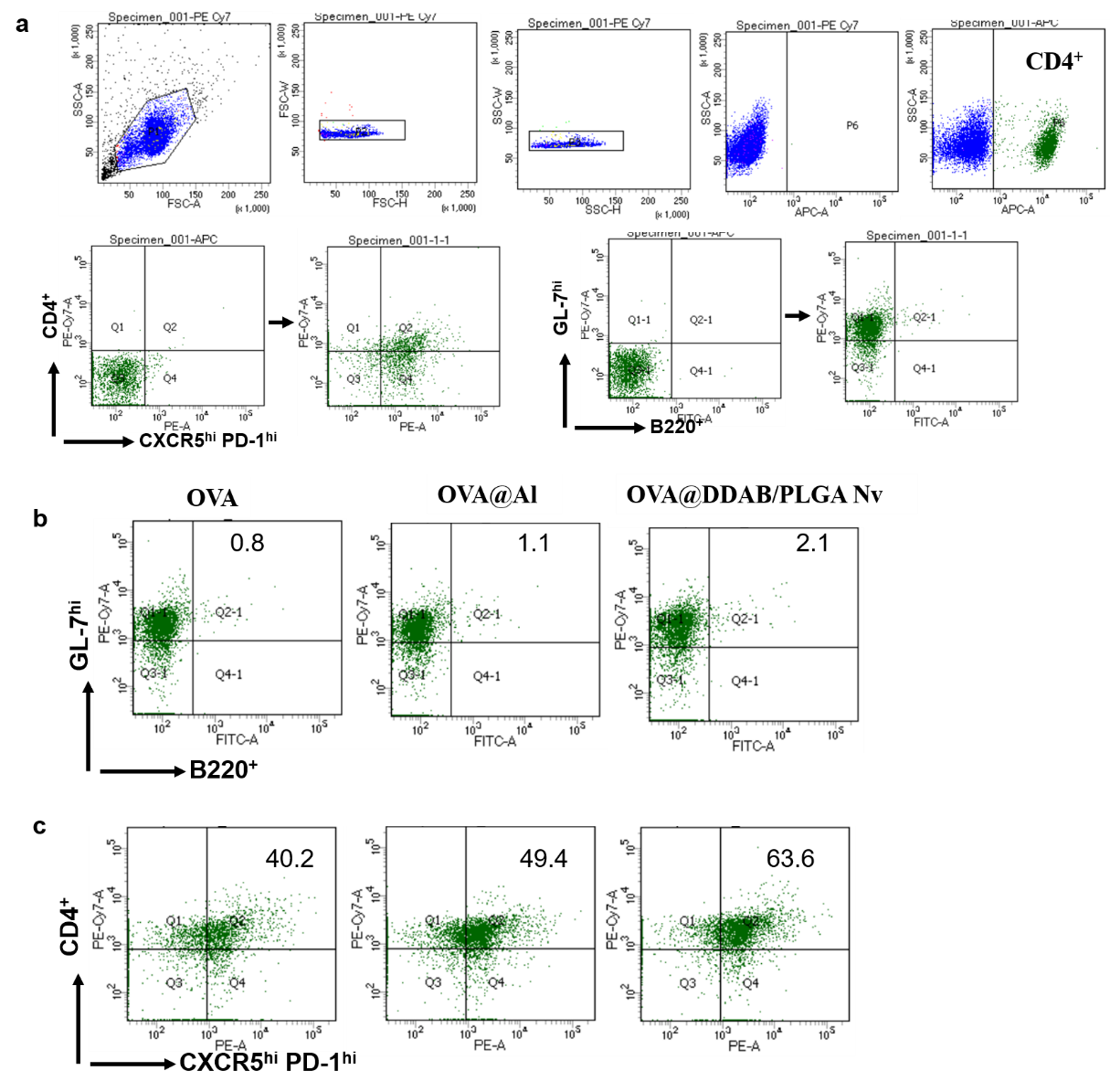


Figure S8. The OVA@DDAB/PLGA Nv induced the formation of germinal centers in draining LNs. (a) The gating strategies of flow cytometry of germinal center and follicular helper CD4^+^ T cells. (b) The count of germinal center (GL-7^hi^ B220^+^ cells) and (c) the follicular helper CD4^+^ T cells (Tfh, CXCR5^hi^ PD-1^hi^ CD4^+^ T cells) in draining LNs were analyzed by flow cytometry.


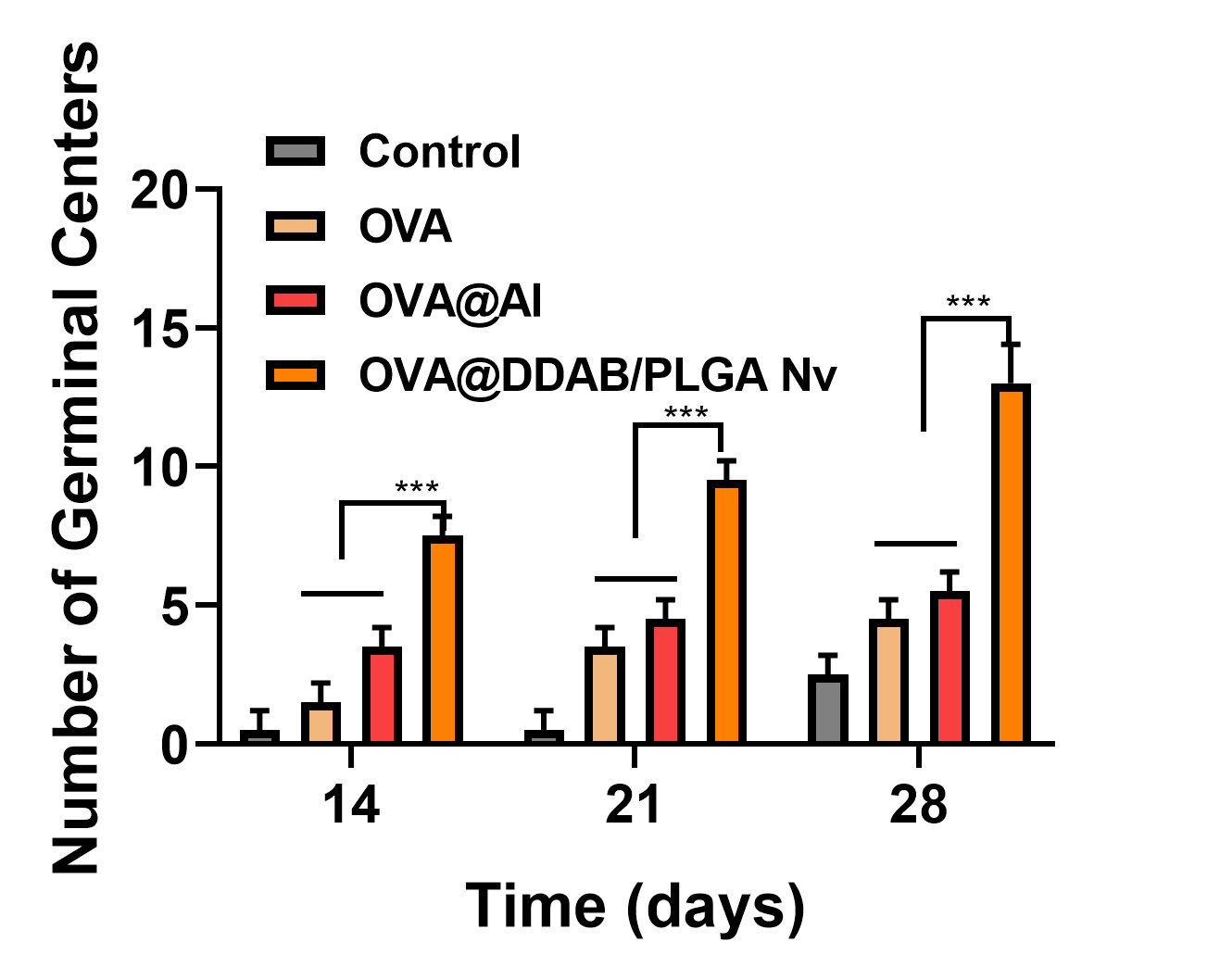


Figure S9. Germinal centers in draining LNs determined by immunohistochemical staining. The data were analyzed by automatic multispectral imaging system (PerkinElmer Vectra II). Three mice were analyzed in every group (n = 3), and data are the mean ± STD and representative of three independent experiments. Differences between two groups were tested using an unpaired, two-tailed Student’s t-test. Differences among multiple groups were tested with one-way ANOVA followed by Tukey’s multiple comparison. Significant differences between groups are expressed as follows: *P < 0.05, **P < 0.01, or ***P < 0.001.


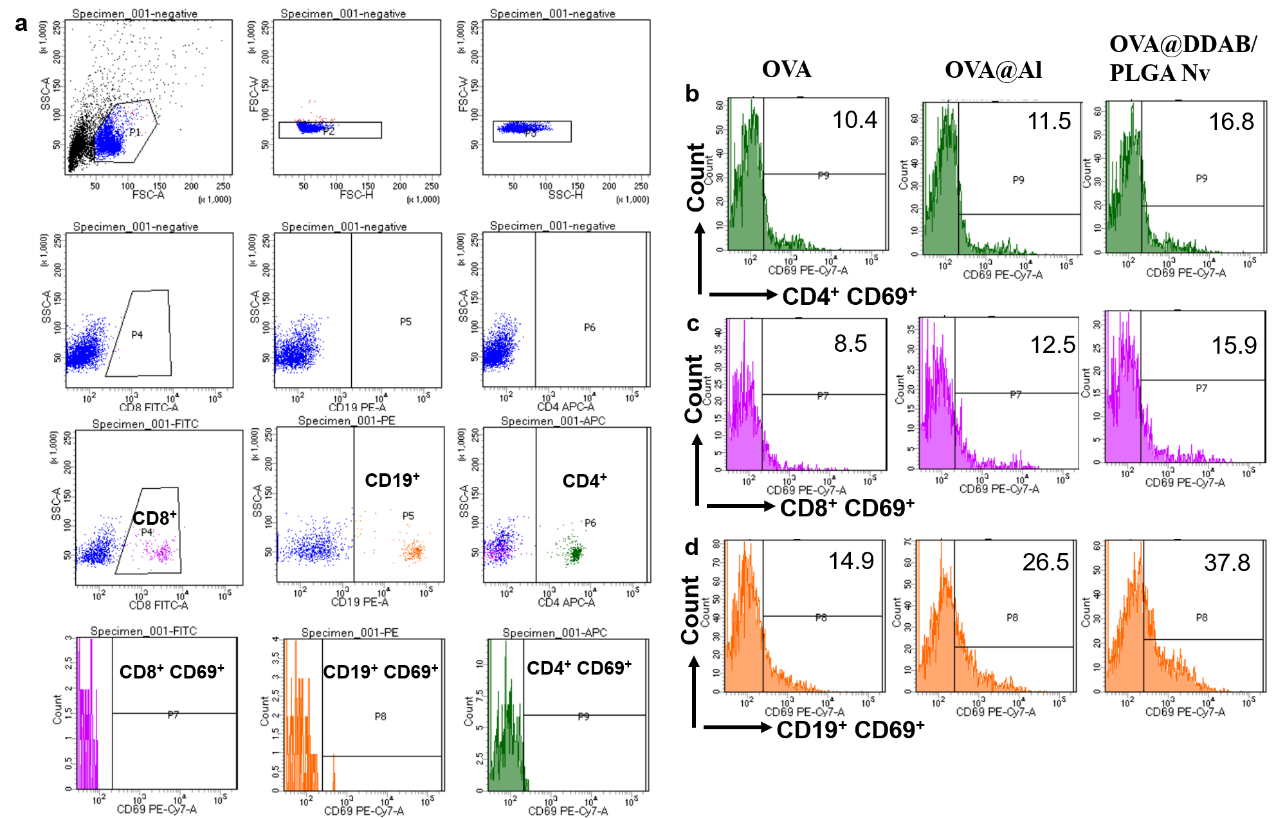


Figure S10. OVA@DDAB/PLGA Nv induced splenocyte activation. (a) The gating strategies of flow cytometry of splenocyte activation. (b, c, d) The activation of CD4^+^ T cells (b) CD8^+^ T cells (c) and B cells (d) from mice after being immunized with different vaccine formulations were measured by flow cytometry.


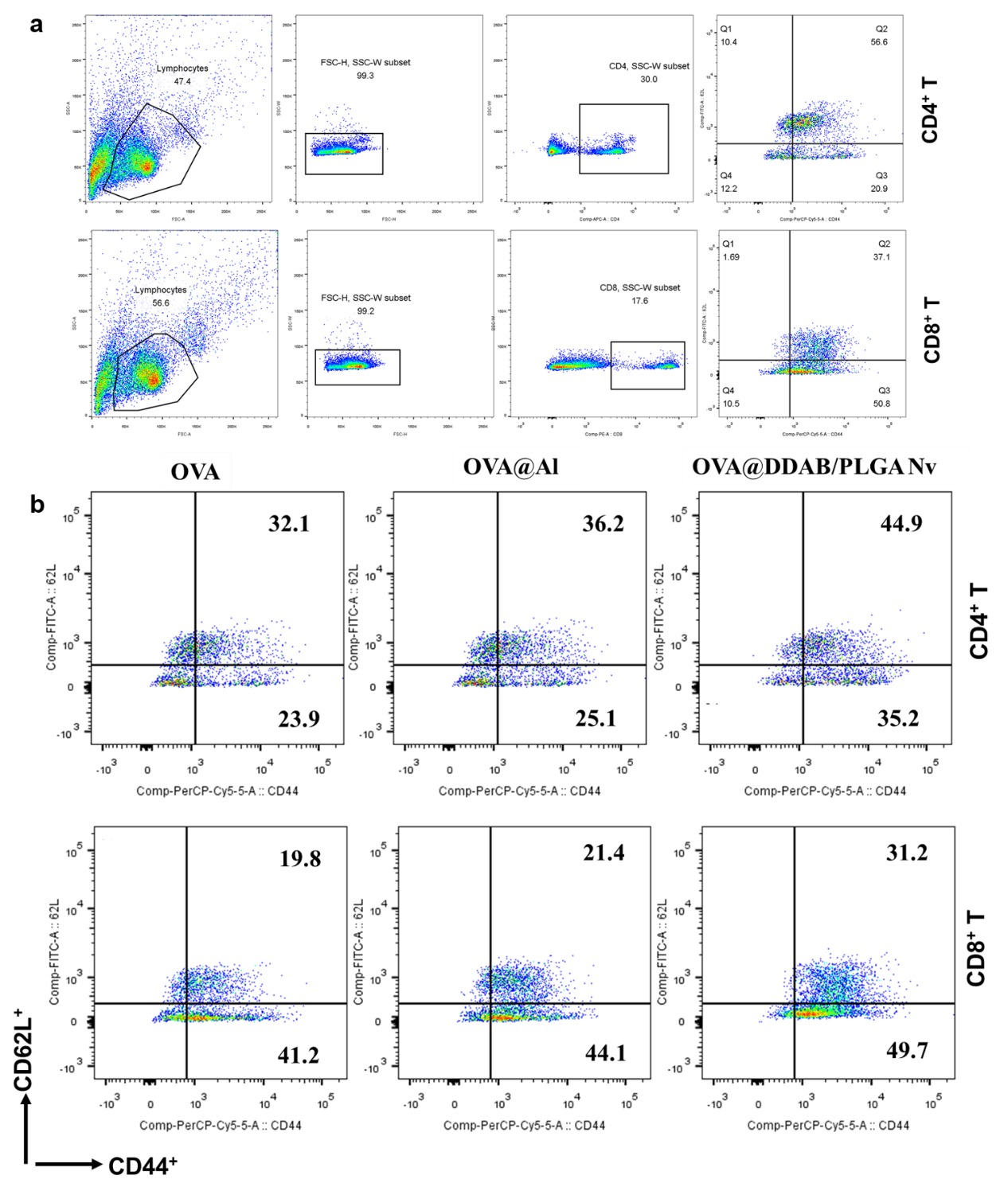


Figure S11. Effects of different vaccines on memory T cell responses. (a) The gating strategies of flow cytometry of memory T cell. (b) Effector memory (CD44^hi^ CD62L^low^) andcentral memory (CD44^hi^ CD62L^hi^) in CD4^+^ and CD8^+^ T cells were measured by flow cytometry.


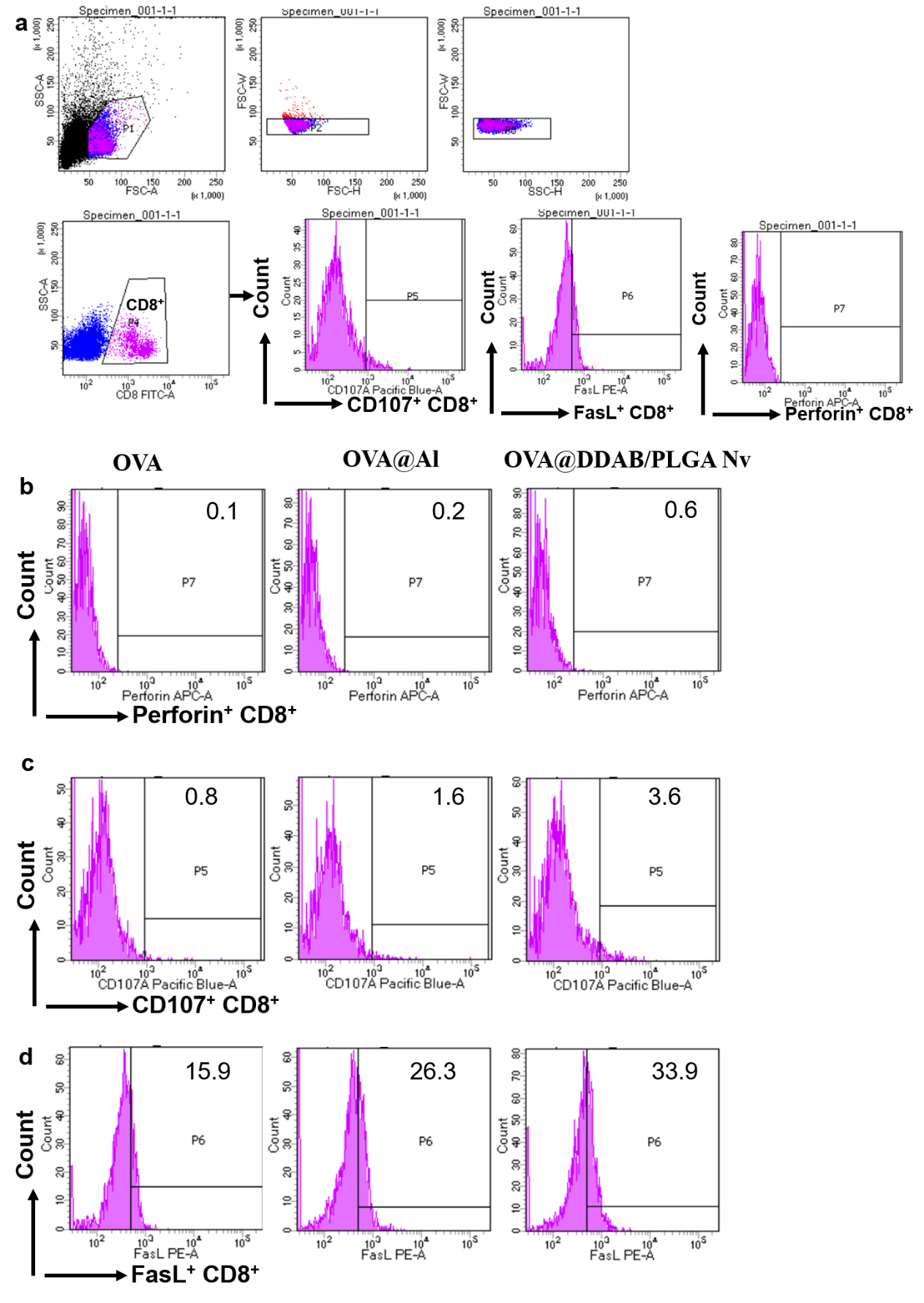


Figure S12. Effects of different vaccines on CTL response. (a) The gating strategies of flow cytometry of CTL cells. The expression of Perforin (b), CD107 (c), and FasL (d) on CD8^+^ T cell in splenocytes were measured by flow cytometry.


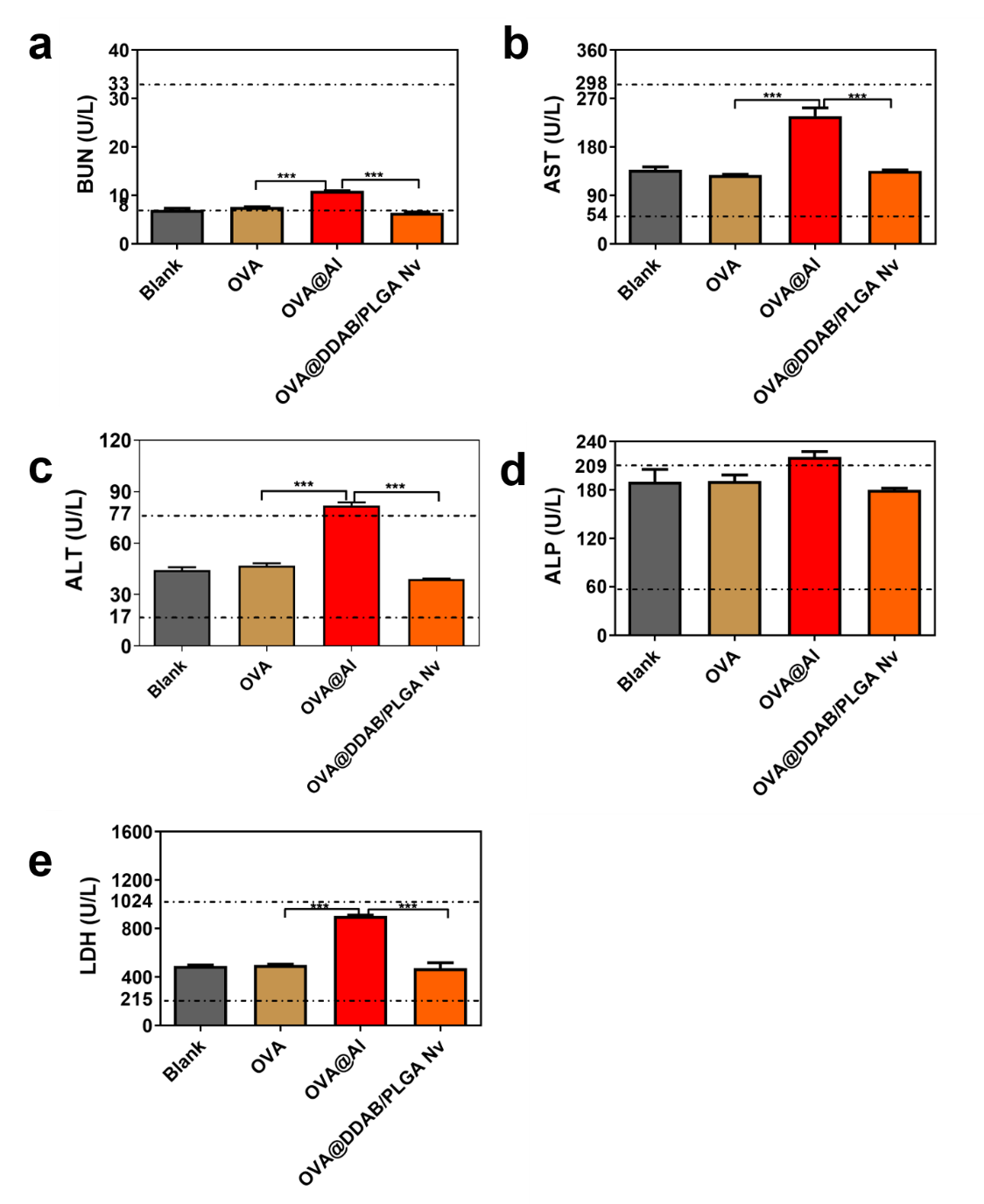


Figure S13. *In vivo* toxicity evaluation of DDAB-PLGA Nv. Hematological analysis of treated mice after 35 days. The range marked by dotted lines represents the normal range of different biosafety indicators. The determination of serum biochemistry of urea nitrogen (BUN) (a), aspartate transaminase (AST) (b), alanine aminotransferase (ALT) (c), alkaline phosphatase (ALP) (d), and lactate dehydrogenase (LDH). Three mice were analyzed in every group (n = 3), and data are the mean ± SEM and representative of three independent experiments. Differences between two groups were tested using an unpaired, two-tailed Student’s t-test. Differences among multiple groups were tested with one-way ANOVA followed by Tukey’s multiple comparison. Significant differences between groups are expressed as follows: *P < 0.05, **P < 0.01, or ***P < 0.001.
